# Enhancing propensity score analysis with data missing not at random: Introducing dual-forest proximity imputation

**DOI:** 10.3758/s13428-026-03024-x

**Published:** 2026-05-29

**Authors:** Yongseok Lee, Walter L. Leite

**Affiliations:** 1 Department of Human Development and Family Science, Purdue University, Hanley Hall (Room 356), 1202 Mitch Daniels Blvd, West Lafayette, IN 47906 USA; 2 School of Human Development and Organizational Studies in Education, University of Florida, Gainesville, FL USA

**Keywords:** Propensity score analysis, Multiple imputation, Missing not at random, Random forests, Dual-forest proximity imputation

## Abstract

Researchers using propensity score analysis (PSA) to estimate treatment effects using secondary data may have to handle data that are missing not at random (MNAR). Existing methods for PSA with MNAR data use logistic regression to model the missing data mechanisms, thus requiring manual specification of functional forms, and are difficult to implement with a large number of covariates. To overcome these limitations, this study proposes alternatives to existing methods by replacing logistic regression with a random forest. Also, it introduces the dual-forest proximity imputation method, which leverages two types of proximity matrices of random forest techniques and incorporates missingness pattern information in each matrix. Results from a Monte Carlo simulation show dual-forest proximity imputation’s enhanced bias reduction with various types of MNAR mechanisms as compared to existing and alternative methods. A case study is also provided using data from the National Longitudinal Survey of Youth (NLSY79).

**Supplementary Information:**

The online version contains supplementary material available at 10.3758/s13428-026-03024-x.

## Introduction

Researchers using propensity score analysis (PSA) to estimate causal treatment effects with observational data frequently encounter issues arising from missing data (Leite et al., 2021). Estimates obtained with PSA may be significantly impacted by missing responses (Little & Rubin, 1987), as the loss of information makes it difficult to accurately estimate propensity scores (PS) and assess the balance in covariate distribution between treatment and control groups (Rubin, 1987; D'Agostino & Rubin, 2000; Mattei, 2009). A few studies have investigated multiple imputation (MI) methods to reduce treatment effect bias in PSA (Hill, 2004; Puma et al., 2009; Tilling et al., 2016; Leyrat et al., 2019; Little & Rubin, 2014; Leite et al., 2021). MI for PSA requires the imputation process to be conducted separately by treatment status (Puma et al., 2009), the specification of a functional form for imputing missing covariates (Little & Rubin, 2014), and the inclusion of the outcome variable in the imputation model (Enders et al., 2016; Graham, 2009).

Data that are missing not at random (MNAR) pose unique challenges for PSA. MNAR is a missing data mechanism where the probability of missingness is driven by either the missing variable itself (e.g., censored) or an unobserved auxiliary variable (Collins et al., 2001; Enders, 2010). PSA with missing data frequently makes the missing at random (MAR) assumption, where the probability of missingness relates to observed data. Under the MAR assumption, PSA must include observed covariates related to missingness in the PS model. In contrast, PSA with the MNAR assumption requires that additional information, such as missing indicators, be included in PSA alongside standard multiple imputations (Enders, 2010; van Buuren, 2018; Beesley et al., 2021).

For PSA with MNAR data, prior studies have proposed the missingness pattern (MP) and multiple imputation with missingness pattern (MIMP) methods, which yield improved estimates under MNAR mechanisms (D'Agostino & Rubin, 2000; Little & Rubin, 2014; Qu & Lipkovich, 2009; Mattei, 2009; Blake et al., 2020). The MP method estimates PS using logistic regression (LR) with covariates available per missingness pattern (MP-LR; Qu & Lipkovich, 2009). The MIMP estimates PS with an LR model that includes pre-imputed covariates and missing indicators (MIMP-LR; Qu & Lipkovich, 2009; Mattei, 2009; Blake et al., 2020). Although previous studies show that MP and MIMP improved treatment estimates in PSA with MNAR data, there are a few shortcomings of using these two methods: First, the imputation process of target confounders in the MP and MIMP methods did not reflect their relationship with treatment assignment and outcome (Enders et al., 2016; Graham, 2009; Puma et al., 2009; Leite et al., 2021). Second, it is hard to specify the nonlinear forms of the imputation models, such as quadratic forms, interactions of observed variables, and missing indicators (Choi et al., 2019) in the MP and MIMP methods. Third, these two methods are likely to experience over-parameterization issues when fitting either the imputation model (McNeish, 2017) or the PS model (Pirracchio et al., 2012) if the data have many confounders. If different types of PS models are fitted on the small number of stratified samples or the PS model implements many confounders with missing indicators, inverse probability of treatment weighting (IPTW), a common approach for implementing PSA, is likely to have some extreme values, introducing treatment effect bias and excessive variance (Leite, 2017; Li & Thomas, 2019).

To alleviate these drawbacks, the random forest (RF) method can provide substantial benefits. RF naturally captures nonlinearities, interactions, and the complex roles of missing indicators without requiring prespecified functional forms (Strobl et al., 2009; Ferri-Garcia & Rueda, 2020). Moreover, by utilizing a proximity matrix, RF-based imputation can directly exploit the distance between observations, including missingness patterns (Tang & Ishwaran, 2017). We propose alternative methods where we replace LR components in MP and MIMP with an RF model, resulting in the MP-RF and MIMP-RF methods, respectively. Furthermore, we build on existing proximity imputation methods and present the dual-forest proximity imputation (DFPI). This new method provides a more robust framework for handling MNAR mechanisms in PSA. We compare DFPI with traditional approaches (i.e., MP-LR and MIMP-LR) and alternative approaches (i.e., MP-RF and MIMP-RF). We also investigate proximity imputation within RF as a pre-imputation step before PS estimation, proposing two variants: the first applies proximity imputation alongside missing indicators (PI-I), whereas the second uses unsupervised RF for proximity imputation (PI-U).

This study is structured as follows: First, we review the definition and assumptions underlying existing methods for MNAR data. Second, we provide alternative versions of existing methods by replacing LR with RF and propose the new DFPI method. Third, we used a Monte Carlo simulation to compare the performance of DFPI with six other imputation methods with PSA, examining the bias of treatment effect estimates and standard errors. Fourth, using an illustrative example with data from the National Longitudinal Survey of Youth 1979 (NLSY79), we demonstrate the DFPI method in comparison with other methods for PSA by comparing common support, covariate balance, and treatment effect estimates.

## Theoretical framework

### Missing data mechanisms

According to Rubin (1987) and Schafer (1997), missing data theory models each individual’s score as comprising an observed value ($${Y}_{observed})$$, missing value ($${Y}_{miss}$$), and a binary missing indicator, $$R$$, where $$R=1$$ if the actual value of *Y* is observed and $$R=0$$ otherwise. The missing indicator ($$R$$) is available information that represents the location of the missing data. In the dataset, the distribution of missing indicators can be explained by a missing data model $$P\left(R|{Y}_{observed},{Y}_{miss},\varphi \right)$$ with parameters $$\varphi$$. The parameter $$\varphi$$ denotes known or unknown quantities (e.g., constant missing probability or coefficients in the missing data model) that define the specific probability distribution of the missingness, allowing researchers to model and infer the nature of the missing data.

Using the general form of the missing data model, we can explain the three types of missing mechanisms. Missing completely at random (MCAR) occurs when $P\left(R=0|{Y}_{observed},{Y}_{miss},\varphi \right)=P(R=0|\varphi )$, indicating that the probability of missingness relies only on $\varphi$ and is independent of both observed and missing values of a certain column. For missing at random (MAR),

$$P\left(R=0|{Y}_{observed},{Y}_{miss},\varphi \right)=P(R=0|{Y}_{observed},\varphi )$$

indicates that the missingness pattern depends on the observed information of the dataset. However, the missing not at random (MNAR) pattern follows

$$P\left(R=0|{Y}_{observed},{Y}_{miss},\varphi \right)$$

which presents the missingness that is explained by the distribution or unobserved information of ${Y}_{miss}$. While the missing data mechanism can be theoretically expressed in general terms, in practice, researchers often do not know the origin of the missingness or the specific probability distribution governing it, as the parameters are typically unknown.

Analysis of MNAR data focuses on the parameters of the missing data distribution, which results from the truncation of the distribution of the variable itself or the effect of an unobserved variable. Consequently,

$$P\left({Y}_{miss}|{Y}_{observed},R=0\right)\ne P\left({Y}_{miss}|{Y}_{observed},R=1\right)$$

The missing data model must contain the parameters of the missing indicator and incorporate information into the substantive analysis model to mitigate the bias of estimates (Enders, 2010). Incorporating the propensity of missingness into the substantive model is the logical foundation of two MNAR models: the selection model (Heckman, 1976) and the pattern-mixture model (Glynn et al., 1986; Little, 1993).

In Heckman’s (1976) selection model, the joint distribution of data and missingness probability, $P\left(Y,R\right)$, is the product of two components of distribution: the conditional probability of missingness given the data ($P\left(R|Y\right)$) and the marginal distribution of data ($P(Y)$):

1 $$P\left(Y,R\right)=P\left(R|Y\right)P(Y)$$

In the context of the equation above, the conditional probability of missingness given the data presents the probability of missing response predicted by observed predictors, potentially related to the missing data mechanism that can be drawn by the regression (e.g., probit). The marginal probability of data indicates the probability of obtaining a response in MNAR, which can be estimated through substantive analysis (e.g., regression) that would have been estimated had there been no missingness in the dataset. The selection model incorporates the dependency of two models, following the bivariate normal distribution of error terms, which has a zero mean vector and a variance–covariance matrix consisting of the error terms of the selection model and the outcome model. Suppose the size of the correlation (covariance) of two error terms is nonzero. In that case, this magnitude quantifies how much the estimates of the substantive regression model reduce the bias from the estimates based on the MAR mechanism.

Although the two-stage selection model helps mitigate the bias of MNAR, it has three disadvantages that can lead to potential bias. First, suppose the selection model fails to capture the hidden relationship (e.g., interaction or nonlinear terms) between covariates. In that case, it may distort the predicted probabilities of response, which can affect the results of the substantive regression model. Second, in model specification, the collinearity of covariates in the selection model and outcome model becomes a source of biased estimates, as the information from the selection model is the explanatory variable in the outcome model. This violates the exclusion-restriction rule, which indicates that the missing response should stem from the probability of missingness itself that MNAR assumed, not from the other factors (e.g., covariates) (Wooldridge, 2010; Puhani, 2000). Third, the parameters estimated in the selection model strongly rely on the bivariate normal distribution. If the shape of the population distribution of outcome is highly skewed or non-normally distributed, substantial bias can be produced (Enders, 2010).

### The pattern-mixture model

The pattern-mixture model (PMM; Glynn et al., 1986; Little, 1993; Rubin, 1987) is a reverse factorization of the joint distribution of selection model. The joint distribution of PMM has the same meaning as that of the selection model, but the roles of the data and missingness are reversed.

2 $$P\left(Y,R\right)=P\left(Y|R\right)P(R)$$

Incorporating the distribution of missingness into the analysis, PMM differs from the selection model in that it stratifies into subgroups based on the similarity of missingness patterns, estimating the outcome model separately for each pattern. The conditional distribution of the data indicates the outcome (substantive) model that is fitted into each missingness pattern, and the marginal distribution of missingness is the proportion of cases with each missingness pattern. Based on Eq. (2), the target of PMM is to obtain a single estimate, averaging the estimates of the outcome model in each pattern, weighed by the respective proportion of missingness pattern.

The critical problem in implementing the PMM process stems from its under-identified data structure due to the inestimable parameters. Specifically, observations lack information on certain variables, depending on the missingness pattern they are in. Therefore, fitting the outcome model with control for all target variables is impossible due to insufficient statistics for the variables. To address the situation, Little (1993) recommended a complete-case missing variable restriction, allowing the inestimable parameters to be transferred from the estimable parameters in the complete-case analysis. With this substitution, sufficient statistics for missing cases and complete cases of variables in MNAR can be calculated and finally weighted by the proportion of missing cases and complete cases. Contrary to the selection model, which depends on the bivariate normal distribution assumption, the PMM is relatively free from the distribution assumption. However, suppose the assumed information of the complete case does not apply to other missingness patterns. In that case, the model fails to reduce the bias of MNAR variables and causes substantial bias even in MAR settings (Demirtas & Schafer, 2003).

### The missingness pattern method

Rosenbaum and Rubin (1984) proposed the MP method for PSA with MNAR data, which applied the logic of PMM with a dataset of missing covariates. Rosenbaum and Rubin (1984) and D’Agostino and Rubin (2000) noted that a generalized PS could be estimated by a separate logit model using the subset of fully observed covariates, depending on each missingness pattern in the large sample. The purpose of this method is to balance the distribution of treatment and control groups in observed covariates and each pattern of missingness. However, as many patterns of missing data involve a few individuals in both the treatment and control groups, it is challenging to apply straightforward PMM in practice. To address these drawbacks, D’Agostino and Rubin (2000) and Mitra & Reiter (2016) proposed the concept of a general location model, which smooths the parameters of the treatment indicator using observed variables across different missingness patterns, employing the expectation–maximization (EM) and expectation–conditional maximization (ECM) algorithms. By using loglinear constraints on categorical variables that include missing indicators, refined parameters offer stable information to estimate PS for each observation.

### Assumptions

For estimating the treatment effect with PSA, four assumptions are required to interpret the treatment effect as a causal effect (Rosenbaum & Rubin, 1984; D’Agostino & Rubin, 2000). First, the consistency assumption states that for each individual $i$, receiving a treatment (${Z}_{i}$) results in the potential outcomes (${Y}_{t}\left(i\right),{Y}_{c}\left(i\right)$). Second, the stable unit treatment value assumption (SUTVA) asserts that the treatment assignment status of an individual $i$ does not affect the potential outcomes of another individual ${i}^{^\prime} \left({Z}_{i},{X}_{i},{Y}_{t}\left(i\right),{Y}_{c}\left(i\right)\perp {Z}_{i}^{^\prime},{X}_{i}^{^\prime},{Y}_{t}\left({i}^{^\prime}\right),{Y}_{c}\left({i}^{^\prime}\right)\right)$. Third, positivity assumption means that the probability of obtaining treatment or control given individual characteristics (${X}_{i}$) is within a boundary of 0 and 1 (${0<P(Z}_{i}=1\left|{X}_{i}\right)<1$). Fourth, the strongly ignorable treatment assignment (SITA) assumes that treatment assignment is independent of the potential outcome status, given that the individual confounders (${Y}_{t}\left(i\right),{Y}_{c}\left(i\right)\perp {Z}_{i}|{X}_{i}$). This also means there are no unmeasured confounders that explain treatment and outcome status. These four assumptions are based on the dataset without missing data, which needs correction to account for missingness.

In the missing data, we can divide a certain covariate, ${X}_{i}$, into two components ${X}_{i}=({{X}_{i}}_{observed},{{X}_{i}}_{miss})$, the observed and missing part of participant $i$ for ${X}_{i}$. The missingness pattern of ${X}_{i}$ can be described as ${R}_{i},$ which has 1 when the elements of ${X}_{i}$ are missing, and otherwise has 0. The SITA assumption can be extended considering this missingness pattern, which is called missingness SITA (mSITA: ${Y}_{t}\left(i\right),{Y}_{c}\left(i\right)\perp {Z}_{i}|{{X}_{i}}_{observed},{R}_{i}$), which accounts for both the observed part and the missingness pattern (Rosenbaum & Rubin, 1984; Mattei, 2009). If we apply the mSITA assumption (Rosenbaum & Rubin, 1984; D’Agostino & Rubin, 2000) to the PSA framework, the PS, $e\left({X}_{i}\right)$, is estimated on each missingness pattern, including only non-missing covariates in that pattern (MPA: missingness pattern approach).

3 $$e\left({{X}_{i}}_{observed},{R}_{i}\right)=P\left({Z}_{i}=1|{{X}_{i}}_{observed},{R}_{i}\right)$$

with the mSITA assumption, the consistent estimator of causal effect by including the information of the MPA requires additional assumptions: conditionally independent treatment (CIT) and conditionally independent outcomes (CIO) assumptions (Mattei, 2009; Blake et al., 2020).

4 $$CIT: {Z}_{i}\perp {{X}_{i}}_{miss}|{{X}_{i}}_{observed},{R}_{i}$$

5 $$CIO: {Y}_{t}\left(i\right),{Y}_{c}\left(i\right)\perp {{X}_{i}}_{miss}|{{X}_{i}}_{observed},{R}_{i}$$

CIT assumption requires that the association between confounder and treatment is absent in the unmeasured subset of ${X}_{i},$ and the CIO assumption indicates that the relationship between potential outcome and confounder is absent in the subset of missing values in ${X}_{i}$. With the mSITA assumption, if either CIT or CIO holds, covariate ${X}_{i}$ does not play a role as a confounder, although it is missing. In other words, it is hard to satisfy these assumptions in practice, in that this assumption underlies the situation in which the missing value must be a different relationship between treatment status and outcome compared to the observed values (Blake et al., 2020). Therefore, these assumptions (mSITA, CIT, or CIO) provide a valid statement supporting the logic of the MPA method in the MNAR missing structure (Rosenbaum & Rubin, 1983; D’Agostino & Rubin, 2000), but they do not fully guarantee validity in MCAR settings (Blake et al., 2020).

#### Variations of the missingness pattern method

There are three variations of the MP method according to the mSITA and CIT (or CIO) assumptions: For the first variation, separate PS models are estimated using the non-missing covariates for each specific missingness pattern (MP: Roseenbaum & Rubin, 1984; D’Agostino, 2001; Qu & Lipkovich, 2009). For the second variation, missing values are re-imputed with a constant value, and imputed values are indicated with missing indicators that are included in the PS model (missing Indicator: D’Agostino, 2001; Choi et al., 2019). The third variation involves PS estimation with covariates and a missing indicator after imputation of missing values, which are replaced by two or more imputed values to represent the uncertainty of the imputed value (MIMP: Hill, 2004; Qu & Lipkovich, 2009; Enders, 2010; van Buuren, 2018). Each of the three types of missingness patterns in PS has its pros and cons compared to the other methods according to the missing mechanisms (Rubin, 1987; Crowe et al., 2010; Qu & Lipkovich, 2009).

The MP method supports the logic of the MPA assumption for MNAR settings and utilizes the information provided by the missingness itself, but it increases the variability in estimated PS, as a small set of stratified samples is used for the estimation. For solving this issue, a Bayesian method and EM algorithm were proposed for estimating the parameters of the PS model (Ibrahim et al., 1999; D’Agostino & Rubin, 2000; D’Agostino, 2001), but these methods are infeasible due to their complexity in the computational process (Qu & Lipkovich, 2009). Imputation of a constant value (0 or single imputation) followed by the inclusion of the missing indicator in the PS model can offer an alternative method that alleviates the difficulties in the MP method in practice (Choi et al., 2019). When data are MNAR, this method outperforms the other methods, with small bias and technical ease in implementing the process (Choi et al., 2019). However, this method leads to substantial bias as the missing part of the variable is substituted for a constant value (Allison, 2001; Groenwold et al., 2012).

### Multiple imputation with missingness pattern

The multiple imputation with missingness pattern (MIMP) method is a hybrid approach that combines MI and the logic of the MP method to address missing data in PSA. The method begins with multiple imputations, which fill in missing values by generating plausible estimates from the posterior predictive distribution, ensuring variability across imputations. Unlike MP methods, however, the MIMP method includes missing indicators as additional covariates in the PS model. These indicators stratify observations based on the individual’s missingness patterns which utilize both observed values and missingness information, providing a more accurate estimation of PS, particularly under MNAR mechanisms (Qu & Lipkovich, 2009; Choi et al., 2019). Based on PS, which is estimated per imputed dataset, the treatment effect and its variances are estimated separately and then combined following Rubin’s rule. A detailed technical description of the MP and MIMP methods is presented in Appendix B and Appendix C, respectively.

To overcome some limitations of the MP method, the MIMP method benefits from the imputation stage by using MI. The MIMP method enhances the realism of the imputed sample during the analysis stage by leveraging MI characteristics, thereby partially elucidating the logic of the MP method (Hill, 2004; Qu & Lipkovich, 2009). By including missing indicators in the PS model, information from both the observed covariate values and the missingness itself (i.e., whether a value is missing) is used when estimating the PS. However, prior studies have noted that while the MIMP method performs well compared to MI without missing indicators under MNAR mechanisms, its advantage diminishes under MAR and MCAR mechanisms, where simpler methods may suffice (Lu & Ashmead, 2018).

According to Qu and Lipkovich (2009) and Choi et al. (2019), the MIMP method is advantageous for obtaining unbiased treatment effects in MNAR in the scenario of no unmeasured confounding and homogeneous treatment effects. However, some of the bias remains in the scenario of heterogeneous treatment effects, noninclusion of outcome in the imputation model, and the existence of unmeasured confounding in PSA. Using a missing indicator in either the imputation model or PS model can lead to unstable treatment effects in the MNAR mechanism, particularly when each missingness pattern is associated with few observations. This can cause non-convergence issues in the MP method and introduce noise in MIMP modeling due to the control of many indicator variables.

Across the MP and MIMP methods, a common critical problem arises in practice when grouping the missingness pattern: using the missingness pattern with small numbers of observations in certain patterns leads to unstable estimates in PS. As the PS model tends to deal with a larger number of covariates than other statistical analyses (e.g., regression, structural equation modeling), finding a homogeneous missingness pattern concerning many covariates in PS might be a difficult task, especially in the fixed sample size. For solving this problem, Qu and Lipkovich (2009) proposed a pooling algorithm to ensure sufficient sample size per group. Based on the sorted number of observations in each missingness pattern, the dissimilarity (Euclidean distance) is calculated for other missingness patterns of small sample size, except for the missingness pattern initially having a large sample size (over 100). Then the pooling process is implemented with the closest missingness pattern until exceeding 100 observations. To maximize similarity, the small missingness pattern is combined with the closest and largest missingness pattern. This method is effective in maintaining the minimum size of missingness patterns to achieve a stable estimator. However, the pooling algorithm's primary drawback is that the threshold for grouping missingness patterns must be determined based on the number of covariates and the distribution of treatment assignment. In the simulation setting discussed by Qu and Lipkovich (2009) and Choi et al. (2019), they pointed out that conditions with a small number of covariates and a large proportion of treated cases provide stable estimates, even when the minimum number of each missingness pattern is 50 observations (less than 100). However, fitting PS fails to converge in situations involving a large number of covariates and a small proportion of treated cases when grouping based on a large minimum threshold (100 observations) per missingness pattern.

### Alternative versions of MP and MIMP using random forests

Random forest (RF) is an ensemble machine learning algorithm used for classification and regression tasks. It operates by constructing multiple decision trees during training, each built from a random subset of the bootstrapped data and features, which promotes diversity among the trees (Breiman, 2001). At each split within a tree, rather than searching over all predictors, RF considers a random subset of size $m$ from the total set of $p$ predictors (i.e., features), where $m$ often defaults to $\sqrt{p}$ and $\mathrm{log}(p)$. This extra randomness of features helps reduce correlation among trees and increase robustness. For the validation process in classification and regression tasks, RF fits the same decision trees on the out-of-bag (OOB) samples, which have observations not included in each bootstrapped sample to estimate the error rate and compute variable importance measures via permutation tests.

To determine the best split in a classification context, RF evaluates different node-impurity measures, aiming to split the data into more homogeneous subsets. Let ${p}_{k}(m)$ denote the proportion of observations in node $m$ that belong to separated class $k$. Generally, impurity criteria include the following:

1. Gini index, $\sum_{k=1}^{K}{p}_{k}(m)(1-{p}_{k}\left(m\right))$, which is minimized at zero if the node contains only one class (pure node);
2. Entropy, $\sum_{k=1}^{K}-{p}_{k}(m)log{p}_{k}(m)$, which measures the “disorder” in a node and increases as the class distribution becomes more uniform;
3. Misclassification error rate, $\frac{1}{{N}_{m}}\sum_{i\in {R}_{m}}I({y}_{i}\ne k(m))$, which is the proportion of observations in node $m$ that do not belong to the node’s majority class $k(m)$.

In addition to these splitting criteria, RF performance depends on several hyperparameters, including the number of trees (*ntree*), the number of features randomly sampled at each split (*mtry*), the maximum depth of each tree (*max_depth*), and the minimum number of samples required to split an internal node (*node size*) (Amusa et al., 2021; Cannas & Arpino, 2019). Fine-tuning these hyperparameters helps prevent overfitting and can significantly improve model performance.

For regression problems, the final prediction is typically the average of all sub-tree predictions (leaf-node’s classification), expressed as

6 $$RF\left(x\right)=\frac{1}{N}{\sum }_{i=1}^{N}{T}_{i}(x)$$

where ${T}_{i}(x)$ indicates the prediction from the *i*th tree, and *N* is the total number of trees. In classification, the output of this algorithm is the mode of the class predictions from all trees. By aggregating the results of multiple trees, random forests reduce the risk of overfitting and improves overall model accuracy and robustness (Strobl et al., 2009). For PS estimation, without missing data, RF has been shown to outperform logistic regression when there are interactions between covariates (Ferri-Garcia & Rueda, 2020). With MNAR data, we argue that integrating RF into MP and MIMP methods can enhance PS estimation by modeling nonlinearities and interactions among covariates and their missing indicators (i.e., missingness patterns).

#### Missingness pattern by random forest

Similar to the principles of the MP and MIMP methods, the extension we propose incorporates the use of RF as a model for PS estimation. In leveraging RF’s capacity to learn nonlinearities and high-order interactions, MP-RF and MIMP-RF explicitly model the joint structure of covariates and missing indicators, attenuating residual links among treatment, outcome, and missingness. This richer propensity surface brings the analysis closer to the conditions required for mSITA, CIT, and CIO, thereby strengthening the causal validity of subsequent effect estimates.

First, the missingness pattern by random forest (MP-RF) method retains the basic structure of the original MP method, following the same analysis stages but enhancing the flexibility and robustness of PS estimation. This is achieved by applying RF to a stratified dataset created by pooling observations with the same missingness pattern. Unlike traditional parametric models (e.g., logistic regression), which can suffer from limitations such as overfitting in high-dimensional datasets that can lead to a lack of common support (Austin, 2011), the MP-RF method leverages the nonparametric and ensemble-based capabilities of RF. These features also allow RF to capture complex relationships and interactions among covariates (Breiman, 2001), making it a powerful and versatile tool for PS estimation. Building on the framework proposed by Qu and Lipkovich (2009), the MP-RF method adheres to its pooling rule for combining missingness patterns. To be specific, the minimum sample size of each cell is 100 observations sharing the same missingness pattern to ensure stable estimation of PS. Each pooled sample within each cell is then used to train the RF model, which computes PS based on the covariates in a homogeneous missingness structure. Due to the sample reduction inherent to training the RF model within each pooled cell, tuning hyperparameters becomes an important step for achieving accurate PS and minimizing biased treatment effects. Following the recommendations from previous studies (Tang & Ishwaran, 2017; Cannas & Arpino, 2019; Amusa et al., 2021), we set the hyperparameters of RF as follows: the number of trees to 500 (i.e., *ntree* = 500) and the number randomly sampled as candidates at each split to the square root of the total number of covariates (i.e., $mtry=\sqrt{p}$). This process is implemented through the *randomForest* package (Liaw & Wiener, 2002) to ensure robust PS estimation.

Second, the multiple imputation with missingness pattern by random forest (MIMP-RF) method builds on the MIMP method by introducing RF as the model for estimating PS, while applying multivariate imputation by chained equations (MICE) to address missing data (Morris et al., 2014; van Buuren, 2018; Leite et al., 2021). In the MIMP-RF method, the first step is to impute missing values through MICE. MICE iteratively estimates missing values by fitting a series of univariate imputation models, one for each incomplete variable given the other variables in the dataset (van Buuren, 2012; Kleinke, 2017; Leite et al., 2021). These univariate models can be tailored to the scale of the covariate: ordinary least squares for continuous variables, logistic regression for binary variables, or other suitable parametric models. Additionally, supervised machine learning models (e.g., classification trees, random forests, super learner) can be employed within the MICE framework to capture nonlinearities and interactions among variables (Morris et al., 2014; van Buuren, 2018; Leite et al., 2021). To address variability in imputations, similar to the MIMP method, 10 imputed datasets are generated through the MICE process^1^ (Enders, 2010). For each dataset, RF is used to estimate PS based on the fully imputed covariates and the inclusion of missing indicators. Similar to the hyperparameter setting of the RF in the MP method, the randomForest package (Liaw & Wiener, 2002) uses 500 trees and the square root of the total number of covariates per splitting node. RF’s ability to handle the high-dimensional data (covariates and their missing indicators) and model complexity ensures that the estimated PS is robust and accurate regarding missingness patterns.

### Proximity imputation in random forest

Proximity imputation using a random forest can effectively address several challenges posed by MP-RF and MIMP-RF when the number of covariates becomes large and the missingness patterns are heterogeneous. By incorporating the proximity measure of RF, it is possible to capture distances between units regarding observed and shared missingness patterns, thus alleviating the risk of forming heterogeneous or under-sampled groups. Likewise, in the MIMP-RF, a PS model with missing indicators can be fitted under high-dimensional data, which can be complex and lead to unstable parameter estimates. Therefore, a pre-imputation process using a proximity-based method can reduce this complexity by imputing values based on similarities in the observed space. In both scenarios, proximity imputation provides a data-driven, flexible pre-imputation mechanism that can mitigate model instability and improve overall estimation under the MNAR mechanism.

Regardless of its relevance to MP-RF and MIMP-RF, we should investigate the impact of the proximity imputation method, which can be pursued independently for MNAR mechanisms. While MP-RF capitalizes on grouping by missingness pattern and MIMP-RF utilizes PS modeling for data augmentation, the proximity-based strategy bypasses these mechanisms by relying on distance within the RF’s leaf structure. As the RF-based distances are computed jointly from confounders, treatment status, missing-data indicators, and outcome, the proximity-based imputation tends to reduce systematic differences in missingness patterns (i.e., the distribution of missing indicators) across treatment arms before any PS modeling. This pre-balancing can relax the risk of violating mSITA and, by reducing residual confounding linked to missingness, make the subsequent CIT and CIO assumptions more tenable than in MP-RF or MIMP-RF. This independence underscores its flexibility: practitioners can incorporate proximity-based imputation before PS estimation, or they can adopt proximity-based imputation exclusively (i.e., use PI to create a complete dataset and then proceed with standard PSA) when traditional methods are deemed unsuitable (e.g., highly sparse missingness-pattern cells) or with overly restrictive specification in the imputation model. Therefore, introducing what the proximity imputation is and how to combine missingness patterns in the proximity matrix in this study highlights an additional pathway for efficiently handling MNAR mechanisms.

In RF, proximity between observations can be measured using a proximity matrix (Breiman, 2001; Tang & Ishwaran, 2017). The $N\times N$ matrix measures the similarity between $N$ observations by tracking the proportion of trees in which pairs of observations are assigned to the same leaf node in the forest. This proportion reflects their proximity (i.e., higher values indicate greater similarity) and is used as weights in the proximity imputation algorithm (Breiman, 2001). For the missing imputation strategy in the PSA, a previous study found that proximity imputation outperformed the traditional MI method in obtaining an unbiased treatment effect (Author, 2024).

In the present study, we combined missing indicators with proximity imputation as a pre-imputation process, implementing two types of proximity imputation that include missing indicators. The first type is general proximity imputation with missing indicators (PI-I), which reflects how similar observations are in terms of their likelihood of the outcome, given their treatment status, confounders, and missingness patterns. In this approach, we derive proximity among observations using supervised RF. The model does not predetermine the influence of treatment status, confounders, or its missingness pattern during the fitting process (presented in Appendix D). The second type involves constructing unrestricted forms of RF by selecting any variables during the sub-selection stage (e.g., randomness of splitting); this requires the use of RF in an unsupervised way (Ishioka, 2012, 2013), decreasing the impact of variable selection in leaf and ancestor nodes (PI-U). Therefore, PI-U captures the overall data structure rather than specifically focusing on how confounders relate to treatment and outcome (Appendix E).

Both PI-I and PI-U leverage RF's flexibility in the presence of missing data. PI-I implements a supervised forest to build proximity ties to the outcome and potentially the treatment indicator, which can be beneficial when the relationship between the outcome and covariates is strong (Tang & Ishwaran, 2017; Shah et al., 2014), but not when the relationship is subtle. Conversely, PI-U uses an unsupervised forest to detect latent structure in covariates with missing indicators but tends to underutilize outcome and treatment information (Ishioka, 2012). Despite these trade-offs, both variations improve upon pure mean-based and hot-deck imputation by modeling intricate relationships among covariates, thereby reducing bias under MNAR assumptions (Hong & Lynn, 2020).

### Dual-forest proximity imputation

The proximity imputation methods using missing indicators, such as PI-I and PI-U, each have limitations in fully addressing the complex interplay between treatment assignment, outcome, and missingness patterns. For instance, PI-I depends on a supervised random forest model, which fixes an outcome as a leaf node, potentially overlooking the difficulties in disentangling the relative influence of treatment, confounders, and missing indicators by prioritizing outcome-related features. PI-U, in contrast, employs an unsupervised approach that captures broad data structures in covariates and missing indicators without explicitly targeting treatment or outcome-relevant relationships; as a result, it can underutilize confounder–treatment–outcome relationships that are most consequential for causal estimation. More broadly, other unsupervised representation-learning approaches, such as principal component analysis (Jolliffe, 2002), autoencoders (Kingma & Welling, 2014), and manifold-learning embedding such as t-distributed stochastic neighbor embedding (t-SNE; van der Maaten & Hinton, 2008), may also capture broad structure in *X*, but they likewise would not explicitly prioritize treatment/outcome relationships unless such information is incorporated in a supervised or semi-supervised learning objective (Bengio et al., 2013). Consequently, these methods can account for complex missing mechanisms when computing proximities between observations, unlike MP or MIMP. However, they have struggled to calculate proximity based on confounders and missing indicators separately for treatment and outcome. In other words, in PI-I and PI-U, confounders and missing indicators are not clearly distinguished according to whether they are most relevant to the treatment assignment process or the outcome process.

To address these challenges of PI-I and PI-U, this study provides a novel method, dual-forest proximity imputation (DFPI), that leverages two RF models to create a comprehensive proximity matrix that captures the proximity of observations concerning both treatment assignment and outcome prediction, while accounting for a “dual-lens” view of both treatment assignment and potential outcomes according to the conceptual definition of confounders (Stuart, 2010). Specifically, by partitioning the treatment-driven and outcome-driven proximity matrix into two separate forests, DFPI can balance missingness patterns conditionally on covariates, thereby alleviating the impact of mSITA violation. This approach achieves exchangeability across treatment groups (CIT) and attenuates direct links between missingness and outcomes (CIO). The DFPI method calculates the proximity between observations using missingness patterns in situations with MNAR mechanisms, and this process of calculating the proximity is based on the definition of a confounder. It can consider the complex functional forms of missing indicators and observed information. Moreover, by utilizing two separate RF models: one for treatment status and the other for outcome prediction, it avoids the over-parameterization that can occur when all variables and interactions attempt to be fitted into a single massive forest model. This approach mitigates the bias of MNAR that arises from unobserved factors linked to missingness, because each forest’s proximity metric encapsulates different facets of the data (Blake et al., 2020). Building on the idea that missing indicators can serve as observable proxies for latent influences related to nonresponse, prior work suggests that incorporating missing indicators, especially with imputation, can reduce bias in MNAR mechanisms when missingness is informative (Choi et al., 2019; Sperrin & Martin, 2020). DFPI operationalizes this idea by averaging treatment- and outcome-focused proximity matrices constructed from covariates and missing indicators. For example, when a covariate is MNAR because individuals with systematically higher (unobserved) values are less likely to be measured, the missing indicator flags this latent subgroup; DFPI then imputes using donors that are similar in missingness patterns, treatment assignment, and outcome, helping reduce bias in subsequent PS weighting.

DFPI is an iterative proximity-based pre-imputation procedure that constructs a “confounder-signified” proximity matrix by combining two proximity matrices from two supervised random forests: one focused on treatment assignment and the other focused on outcome prediction. The dual-forest matrix construction is intended to define similarity between units through two complementary lenses, treatment and outcome, while explicitly incorporating missing indicators into the proximity computation. The specific DFPI steps are described below:

Let ${X}_{ij}$ denote an $n\times p$ confounder matrix containing missing values, where $i=\mathrm{1,2},\dots ,n$ indicates observations and $j=\mathrm{1,2},\dots , p$ indicates the number of confounders. Let ${S}_{ij}$ be the corresponding missing indicators, where ${S}_{ij}=1$ if ${X}_{ij}$ is missing and ${S}_{ij}=0$ otherwise. Let ${T}_{i}$ be the binary treatment indicator (${T}_{i}\in \{\mathrm{0,1}\}$) and ${Y}_{i}$ the observed outcome variable.

#### Step 1 (initialization)

Because random forests require a complete predictor matrix, DFPI starts by creating an initial complete dataset ${X}^{(0)}$ using a simple strawman imputation (e.g., replacing each missing ${X}_{ij}$ with column median for continuous variables). This process provides stable starting values to compute proximities and to iterate toward proximity-weighted imputations.

#### Step 2 (dual supervised forests and proximity matrices)

At iteration $m$, construct an augmented predictor matrix ${X}^{*(m)}=[{X}^{(m)}|S]$ that concatenates the current complete confounder matrix and the missing indicators. DFPI then fits two random forests using the same predictors, ${X}^{*(m)}$:

- A treatment-prediction forest

7 $${\widehat{T}}_{i}={f}_{RF,T}({X}^{*(m)})$$

and

(2b) An outcome-prediction forest

8 $${\widehat{Y}}_{i}={f}_{RF,Y}({X}^{*(m)})$$

For each forest, DFPI constructs an $n\times n$ proximity matrix by counting how frequently a pair of observations falls in the same terminal node across trees. Let ${B}_{T}$ and ${B}_{Y}$ be the number of trees in the treatment and outcome forests, respectively, and let ${{\ell}}_{T,b}(i)$ and ${{\ell}}_{Y, b}(i)$ denote the terminal node (leaf) assignment of observation $i$ in tree $b$. The proximity indices are computed as proportions:

9 $${M}_{T}^{\left(m\right)}\left(i,{i}^{`}\right)=\frac{1}{{B}_{T}}\sum_{b=1}^{{B}_{T}}1\{{{\ell}}_{T,b}\left(i\right)={{\ell}}_{T,b}({i}^{`})\}$$

10 $${M}_{Y}^{\left(m\right)}\left(i,{i}^{`}\right)=\frac{1}{{B}_{Y}}\sum_{b=1}^{{B}_{Y}}1\{{{\ell}}_{Y,b}\left(i\right)={{\ell}}_{Y,b}({i}^{`})\}$$

These proximity values lie in the range [0,1], with larger values indicating that two units are more similar with respect to the forest’s prediction task given ${X}^{*(m)}$.

#### Step 3 (confounder-signified proximity matrix)

DFPI combines two proximity matrices by element-wise averaging to obtain the confounder-signified proximity matrix:

11 $${{{M}}}_{{{C}}{{S}}}^{\left({{m}}\right)}=\frac{1}{2}({{{M}}}_{{{T}}}^{\left({{m}}\right)}+{{{M}}}_{{{Y}}}^{\left({{m}}\right)})$$

In practice, DFPI sets the diagonal entries to zero (${M}_{CS}^{\left(m\right)}\left(i,{i}^{`}\right)=0$) so that each observation is imputed using information from other observations rather than itself.

#### Step 4 (proximity-weighted imputation)

DFPI imputes each missing value in the confounder using a proximity-weighted donor from ${M}_{CS}^{\left(m\right)}$. For each observation $i$, define normalized proximity weights over all donors $i\ne {i}^{`}$ as

12 $${{{\omega}}}_{{{i}}{{i}}`}^{({{m}})}=\frac{{{{M}}}_{{{C}}{{S}}}^{\left({{m}}\right)}({{i}},{{{i}}}^{^\prime})}{\sum_{{{k}}\ne {{i}}}{{{M}}}_{{{C}}{{S}}}^{\left({{m}}\right)}({{i}},{{k}})}$$

For continuous confounder ${X}_{i`j}$, if ${X}_{ij}$ is missing, the updated imputed value is computed as a proximity-weighted mean:

13 $${X}_{ij}^{\left(m+1\right)}=\sum_{i`\ne {{i}}}{{{\omega}}}_{{{i}}{{i}}`}^{({{m}})}\cdot{X}_{i`j}^{\left(m\right)}$$

when a donor ${X}_{i`j}$ is originally missing, its current value ${X}_{i`j}^{\left(m\right)}$ is used, which is why initialization in step 1 and iterative updating are required. For a categorical confounder (e.g., binary), DFPI uses proximity-weighted voting by response category. If we define ${C}_{j}$ as a set of categories of variable $j$, the proximity-weighted donor of each category (${c\in C}_{j}$) can be calculated:

14 $${{{\omega}}}_{ij}^{\left(m\right)}(c)=\sum_{i`\ne {{i}}}{{{\omega}}}_{{{i}}{{i}}`}^{\left({{m}}\right)}\cdot 1\{{X}_{i`j}^{\left(m\right)}=c\}$$

To stabilize category assignment in finite samples (especially when categories are imbalanced), DFPI can additionally weight these proximity-based supports by marginally observed category proportion ${p}_{j}(c)$ that is computed from non-missing values of ${X}_{i`j}$, and then assign the category with the largest weighted score:

15 $${X}_{ij}^{\left(m+1\right)}=arg\underset{{c\in C}_{j}}{\mathit{max}}[{p}_{j}(c)\cdot {{{\omega}}}_{ij}^{\left(m\right)}(c)]$$

For binary variables, this reduces to comparing the scores for $c=0$ and $c=1$ and imputing the category with the larger score.

#### Step 5 (iteration and stopping rule)

Steps 2–4 are repeated using the updated dataset ${X}_{ij}^{\left(m+1\right)}$ until the relative difference of the dataset between the newly imputed and the previous one reaches a small relative difference (e.g., $<{10}^{-5}$) based on the change in imputed cell or until the predetermined maximum number of iterations is reached. The final complete confounder matrix is then used as an imputed dataset for PSA.

## Monte Carlo simulation

The Monte Carlo simulation aims to investigate how different strategies for the MPA method using RF perform under the MNAR mechanism. Specifically, we assess their performance with respect to the simulation outcomes. As we mentioned in the previous section, the different strategies commonly use the missingness pattern (i.e., the configuration of missing indicators that defines a subgroup of observations) and apply the logic of RF to the imputation process or PS model reflecting a specific missingness-pattern group. The simulation study had two goals. First, we aimed to benchmark the proposed DFPI method against existing approaches (MP-LR, MIMP-LR) and RF-based extensions (MR-RF, MIMP-RF, PI-I, PI-U) in terms of the relative bias of the average treatment effect (ATE), its standard error, and mean squared error (MSE) under MAR and six types of MNAR mechanisms. Second, we examined how design factors (number of covariates, sample size, percentage of missingness, and six types of missing mechanisms) moderate performance differences across the seven methods (Table 1).

**Table 1:** Models of comparisons

Methods	MPA	(1)	(2)	(3)	(4)	(5)	(6)
MP/MIMP	MP-LR	None	O	X	X	Logistic	O
	MIMP-LR	MICE-PMM	X	X	O	Logistic	X
	MP-RF	None	O	X	X	RF	O
	MIMP-RF	MICE-PMM	X	X	O	RF	X
Pre-imputation	PI-I	RF	X	O	X	Logistic	X
	PI-U	RF	X	O	X	Logistic	X
	DFPI	RF	X	O	X	Logistic	X

(1) Imputation model, (2) sub-sampling by missingness patterns, (3) missing indicators in imputation model, (4) missing indicators in PS model, (5) PS model, (6) pooling missingness patterns

### Method

This study evaluated RF methods to handle missing data following MAR and different subtypes of MNAR mechanisms in PSA through a Monte Carlo simulation.^2^ The study follows the study design in Leite et al. (2021) and Choi et al. (2019), reflecting four types of missing mechanisms: MAR, MNAR-variable itself, MNAR-unobserved variable, and MNAR-interactions.

For generating the treatment assignment condition in the simulated data, the following population logistic regression model was used:

16 $$\mathrm{logit}\left({Z}_{i}=1|X\right)= {\beta }_{0}+ {\beta }_{1}{X}_{1i}+ {\beta }_{2}{X}_{2i}+ \cdot\cdot\cdot+ {\beta }_{k}{X}_{ki}$$

17 $${Z}_{i}=1 if {logit (Z}_{i})>0, else {Z}_{i}=0$$

and

where ${Z}_{i}$ is the treatment indicator, and the $X$’s are true confounders, where half of the covariates are binary and the other half are continuous ones for the real study design. When generating covariates, elements of the population correlation matrix^3^ were taken from the results of the illustrative example (NLSY79 dataset). Covariates were generated using a multivariate standardized normal distribution. ${\beta }_{0}$ is the intercept, which indicates the proportion treated when covariates are zero, and the coefficient vector of (${\beta }_{1}, {\beta }_{2}, \cdot\cdot\cdot ,{\beta }_{k})$ represents the effects of covariates. Following the setting of Leite et al. (2015) and Choi et al. (2019), the population proportion treated was set to 0.2. Also, we selected the coefficient vector (${\beta }_{1}, {\beta }_{2}, \cdot\cdot\cdot, {\beta }_{k})$ from a uniform distribution in the range of −0.4 to 0.4 to obtain a target pseudo-*R*^2^ (McKelvey & Zavoina, 1975).

The population outcome model was specified as

18 $${Y}_{i}= {\pi }_{0}+ {\pi }_{1}{X}_{1i}+ {\pi }_{2}{X}_{2i}+ \cdot\cdot\cdot +{\pi }_{k}{X}_{ki}+\delta {Z}_{i}+{e}_{i}$$

where

${Y}_{i}$: is the outcome,
${\pi }_{0}$: is the intercept,
$\delta$: is the population treatment effect, and
${e}_{i}$: is the residual of the outcome model that will be generated from standard normal distributions.

The coefficient vector (${\pi }_{1}, {\pi }_{2},\cdot\cdot\cdot,{\pi }_{k})$ was selected from a uniform distribution in a range of 0.1–0.8 to obtain a target population *R*-squared. The population pseudo-*R*^2^ for the treatment assignment model and population *R*^2^ for the outcome model were set to 0.2 to obtain a substantial degree of selection bias (Leite et al., 2021).

### Manipulated conditions

Six conditions were manipulated in the study:

1. Number of confounders,
2. Sample size,
3. Proportion of treated cases,
4. Percentage of missingness in covariates,
5. MAR and five subtypes of MNAR mechanisms, and
6. Eight types of MPA methods for obtaining PS.

The number of confounders was chosen based on previous simulation studies: The number of covariates specified in Qu and Lipkovich (2009), Coffman et al. (2020), and Leite et al. (2021) was 12, 10, and 10 or 20. We chose to simulate 10 and 20 confounders, which were similar to the 25th percentile and median among the distribution of the number of covariates from the review of studies (Thommes & Kim, 2011). The two sample sizes were 500 and 2,000, which was similar to various applications of PSA (Thoemmes & Kim, 2011; Leite et al., 2015; Leite et al., 2021). For the proportion of treated cases, 20% of treated cases was specified based on an example using data from the NLSY79 presented in Leite (2017) and Leite et al. (2021). Following Leite et al. (2021) and Choi et al. (2019), the percentages of missing data were set to 10%, 30%, and 50%. Missing proportion and sample size are critical factors for capturing missingness patterns (Qu & Lipkovich, 2009). Hence, we expected that the variation of these conditions would affect the performance of the MPA approach.

In generating non-ignorable missing data mechanisms, with the MAR mechanism, this study crossed Gomer and Yuan’s (2021) focused-diffuse MNAR taxonomy with Enders’s (2010) direct–indirect MNAR distinction and retained a benchmark MAR condition. The resulting typology comprises six mechanisms:

- MAR,
- Focused-direct MNAR (FD-MNAR),
- Focused-indirect MNAR (FI-MNAR),
- Diffuse MNAR (DF-MNAR),
- Diffuse MNAR interaction 1 (DF-MNAR-INT1), and
- Diffuse MNAR interaction 2 (DF-MNAR-INT2).

This comprehensive classification guided the generation of the missing data conditions analyzed herein (Enders, 2010; Gomer & Yuan, 2021). According to these studies, the conditional distribution of missing values under MNAR mechanisms was a joint effect involving the targeted missing variable(s) (${Y}_{miss}$) and either observed variables (${Y}_{obs}$) or unobserved auxiliary variables, or interactions between these variables. We present the missing process function of a subtype of MNAR via conditional distribution as follows:

19 $$f\left(M|{Y}_{obs},\psi \right) \forall\ { Y}_{miss},\psi\ (\mathrm{MAR})$$

20 $$f\left(M|{ Y}_{miss}, \psi \right) \forall\ { Y}_{miss},\psi\ (\mathrm{Focused}- \mathrm{MNAR})$$

21 $$f\left(M|{Y}_{obs},{Y}_{miss}, \psi \right) \forall\ {Y}_{miss},\psi\ (\mathrm{Diffuse}- \mathrm{MNAR})$$

$${M}$$: *Missing indicator,*
$$Y=\left({Y}_{obs},{Y}_{miss}\right)$$: *Data matrix,*
$$\psi$$: *Missing parameters*

Equation (19) describes the conditional distribution of the MAR mechanism, which highlights that missingness does not depend on the missingness in ${Y}_{miss}$ after controlling observed values ${Y}_{obs}$ and missingness parameter $\psi$. Extending beyond the MAR mechanism, Eq. (20) depicts a focused MNAR scenario wherein missingness depends exclusively on the targeted missing variables themselves or an unobserved auxiliary variable, independent of any observed variables. If the focused MNAR mechanism arises from an unobserved auxiliary variable (e.g., indirect MNAR), this variable directly influences the probability of missingness for the targeted variable. Equation (21) shows the conditional distribution of diffuse MNAR, where missingness is diffused across ${Y}_{obs}$ and ${Y}_{miss}$ with missing parameter $\psi$ (Gomer & Yuan, 2021). In other words, this diffuse MNAR depends simultaneously on both observed and missing values in target variable, involving complex interactions. Therefore, the diffuse MNAR can be viewed as an extension of the MAR mechanism, where the missing distribution is partially influenced by observed (or unobserved) variables while also explicitly depending on the missing values of target variable. Therefore, the simulation setting should recognize that the probability of missingness of MNAR is not independent of the MAR to present the whole subtypes of MNAR mechanisms. In the basic setup for generating missingness in the confounder, we fixed the first confounder (${X}_{1}$) to be fully observed in all missing mechanisms. Missing indicators were generated for the remaining confounders (from ${X}_{2}$ to ${X}_{p}$; $p=10 \ or\ 20$) according to the target mechanisms (i.e., MAR and five subtypes of MNAR). Therefore, each simulated dataset contained missing values in ($p-1$) confounders. The percentage of missingness (10%, 30%, or 50%) denotes the targeted marginal rate of missing values in ($p-1$) confounders, rather than the proportion of confounders that are missing:

1. For generating the MAR condition, the missing indicator of other covariates (${R}_{k}$) was predicted by the first confounder $\left({X}_{1}\right)$ that does not have missing values:

$$logit({R}_{k})= {\gamma }_{0}+ {\gamma }_{1}{X}_{1}+{\varepsilon }_{i}$$

(2) For simulating the focused-direct MNAR setting (FD-MNAR), generating the missing indicator should be a conditional distribution of the target variable itself $\left({X}_{k}\right)$:

$$logit({R}_{k})= {\gamma }_{0}+ {\gamma }_{1}{X}_{k}+{\varepsilon }_{i}$$

(3) For the focused-indirect MNAR mechanism (FI-MNAR) that results from an unobserved variable, the imputation model controls an unobserved variable assuming normal distribution, ${X}_{k+1}^{\ast}\sim N(\mathrm{0,1})$.

This variable was not used for the PS or outcome model:

$$logit({R}_{k})= {\gamma }_{0}+ {\gamma }_{1}{X}_{k+1}^{\ast}+{\varepsilon }_{i}$$

(4) For simulating diffuse-MNAR (DF-MNAR), the missing indicator of a certain variable is affected not only by the variable itself but also by other observed variables in the dataset:

$$logit({R}_{k})= {\gamma }_{0}+ {\sum }_{k=1}^{n}{\gamma }_{k}{X}_{k}+{\varepsilon }_{i}$$

(5) For simulating the diffuse-MNAR mechanism that occurs as a result of the interaction between the unobserved and observed parts of the confounders in the dataset (DF-MNAR-INT1), the imputation model is set to have an interaction between the observed ones and the target MNAR covariate:

$$logit({R}_{k})\ {\gamma }_{0}+{\gamma }_{1}{X}_{1}+ {\gamma }_{2}{X}_{k}+{\gamma }_{3}\left({X}_{1}\times {X}_{k}\right)+{\varepsilon }_{i}$$

(6) Finally, to reflect the interaction of the unobserved auxiliary variable and observed covariate in the imputation model (DF-MNAR-INT2), we can follow the functional form of

$$logit({R}_{k})= {\gamma }_{0}+ {\gamma }_{1}{X}_{1}+{\gamma }_{2}{X}_{k+1}^{\ast}+{\gamma }_{3}({X}_{1}\times {X}_{k+1}^{\ast})+{\varepsilon }_{i}$$

In these population imputation models, ${\gamma}_{0}$ is the target percentage of missingness in the data, and the slopes (from ${\gamma }_{1}$ to ${\gamma}_{4}$) are randomly generated from a uniform distribution from 0.1 to 0.5 (Sperrin & Martin, 2020; Leite et al., 2021; Rockenschaub et al., 2024). Residual (${\varepsilon }_{i}$) follows the logistic distribution with a location parameter of 0 and scale parameter of 1. The outcome ${R}_{k}$ was categorized as 0 if $\mathrm{logit}({R}_{k})$ is less than 0, and 1 otherwise.

For each combination of the manipulated 504 conditions, *number of covariates* (2) $\times$ *sample size* (2) $\times$ *percentage of missingness* (3) $\times$ *missing mechanisms* (6) $\times$ *MPA methods in PS* (7), 1,000 datasets are simulated using R software.

### Analysis of the simulated datasets

For each simulated dataset, seven types of MPA methods (MP-LR, MIMP-LR, MP-RF, MIMP-RF, PI-I, PI-U, and DFPI) were applied. Following the suggestions of Hill (2004), Leyrat et al. (2019), and Leite et al. (2021), the outcome variable was included in all imputation models, including those with covariates and the treatment variable, except for PI-I. To prevent the convergence problem in the PS estimates of the MP and MIMP methods, the minimum number of observations per missingness pattern was set to 100. To clarify how missingness patterns and pooling behaved across manipulated conditions, we report the number of distinct missingness patterns prior to the pooling process (Appendix F) and post-pooling diagnostics after applying the minimum cell size threshold of 100 (Appendix G) in the supplementary materials. For MIMP methods, as we used multiple imputation through the *mice* package^4^ (van Buuren & Groothuis-Oudshoorn, 2011), ATE and its standard error were obtained by pooling 10 imputed datasets following Rubin’s rule (Rubin, 1987). Also, PI-I was obtained using the randomForest packages (Liaw & Wiener, 2002). In contrast, PI-U and DFPI methods require a specific function that incorporates the randomForest package, developed by the author.

For each imputed dataset, except for MP-RF and MIMP-RF, the logistic regression was used to estimate PS with the missing indicators, following Thommes and Kim’s review (2011) and Leite et al. (2021). PS weighting was used to estimate the average treatment effect (ATE; Stuart, 2010). The PS weight for the ATE is

22 $${w}_{i}\left(ATE\right)=\frac{{Z}_{i}}{{e}_{i}\left(X\right)}+\frac{1-{Z}_{i}}{1-{e}_{i}\left(X\right)}$$

where ${e}_{i}\left(X\right)$ is the estimated PS. Using this weighting, the ATE can be estimated as the difference between the weighted means of the outcome of the treated and untreated groups (Schafer & Kang, 2008). The standard error of treatment effect estimates was obtained by Taylor series linearization using the *survey* package in R (Heeringa et al., 2010; Lumley, 2004).

### Analysis of simulation outcomes

The study compared seven types of MPA methods concerning the relative bias of the ATE and its standard error (Hoogland & Boomsma, 1998; Bandalos & Leite, 2015) as simulation outcomes. The relative bias of the ATE was calculated using the equation

$$RB\left(d\right)\frac{1}{R}\sum_{r=1}^{R}\frac{\overline{{d }_{r}}-\delta }{\delta },$$

where $\overline{{d }_{r}}$ indicates the average of the ATE in the replication *r,* and $\delta$ is the population treatment effect, which is fixed at 0.5. Following Hoogland and Boomsma (1998), we considered bias less than 0.05 in absolute value to be acceptable. The relative bias of standard errors is

$$RB\left(SE\left(d\right)\right)=\frac{\left[\left(SE({d}_{i})-SD\left({d}_{i}\right)\right)/SD\left({d}_{i}\right)\right]}{\mathrm{R}}$$

where $SE({d}_{i})$ is the estimated standard error across iterations of a condition, and $SD\left({d}_{i}\right)$ is the actual standard deviation of treatment effect estimates, which is the empirical standard error. Acceptable relative bias of standard error of the ATE is equal to or less than 0.1 (Hoogland & Boomsma, 1998). We also computed the mean squared error (MSE) of the ATE as a summary of accuracy that incorporates both bias and sampling variability. For each condition, MSE was estimated across replications as

$$MSE(d) = \frac{1}{R}\sum_{r=1}^{R}{\left(\overline{{d }_{r}}-\delta \right)}^{2}$$

The MSE evaluates how much the estimates vary around the true value, with a smaller MSE indicating a more accurate estimator (Yuan et al., 2015).

For the interpretation of results, this study used a mixed-design analysis of variance (ANOVA) for all simulation outcomes. For each of the 504 design cells, we first computed the mean relative bias of the ATE, its standard error, and MSE across the 1,000 replications. These cell-level summaries served as the dependent variables in the mixed-design ANOVA that we used to describe which conditions differentiated the pattern of the bias and MSE. The between-factors in this mixed-design ANOVA are (1) the number of covariates, (2) sample size, (3) percentage of missingness, and (4) the MAR and MNAR missing mechanisms and the seven missingness pattern approaches (MPA) and position of the parameters formed within cell factors. Following the recommendations for Monte Carlo studies (Hoogland & Boomsma, 1998; Bandalos & Leite, 2015), we focused on effect size measured, i.e., generalized eta-squared (${\upeta }_{G}^{2}$; Olejnik & Algina, 2003) or proportion of effect variance (PEV; Aydin et al., 2016), rather than on the significance test. The generalized eta-squared indicates the proportion of total variance in the outcome (including residual variance across Monte Carlo replications) accounted for each effect:

$${\eta }_{G}^{2}\left(effect\right)=\frac{S{S}_{effect}}{S{S}_{effect}+S{S}_{error}+S{S}_{other}}$$

In contrast, the proportion of effect variance is defined as

$$PEV\left(effec{t}_{i}\right)=\frac{S{S}_{effect\left(i\right)}}{{\sum }_{K}S{S}_{effect\left(k\right)}},$$

where the denominator represents the sum of squares of all ($K$) modeled effects and excludes the error term. Therefore, the $\mathrm{PEV}$ reflects the relative importance of each design factor among the systematic effects. Because residual variance can be large in Monte Carlo studies, ${\upeta }_{\mathrm{G}}^{2}$ can be very small even when the $\mathrm{PEV}$ indicates that a factor explains a nontrivial share of the effect variance (Aydin et al., 2016). Reporting both protects from overemphasizing an effect just because it dominates within the set of factors (e.g., high $\mathrm{PEV}$ and very small ${\upeta }_{\mathrm{G}}^{2}$) or underemphasizing an effect that is modest in absolute terms but clearly the main driver among the manipulated conditions. Following Leite et al. (2021) and Lee & Leite (2024), we focused on conditions with ${\upeta }_{\mathrm{G}}^{2}\ge 0.01$ or $\mathrm{PEV}\ge 0.01$ when interpreting design-factor influences. The purpose of this mixed-design ANOVA was not to declare a single “best” method, but to identify which design factors and interactions produced meaningful changes in bias across methods. Therefore, the outcomes of the mixed-design ANOVA were the deviation of the estimated treatment effect from the population treatment effect over the population treatment effect ($(\overline{{d }_{r}}-\delta )/\delta$), and deviation of the estimated standard error of replication *r* from the empirical standard error over the empirical standard error $\left(\left(SE({d}_{r})-SD\left(d\right)\right)/SD\left(d\right)\right)$. Method-specific performance, with particular emphasis on DFPI, was then evaluated using our a priori thresholds for acceptable bias $\left(\left|RB(d)\right|<.05,\left|RB(SE\left(d\right))\right|<.10\right)$ based on meaningful factors.

### Relative bias of ATE

The bias of the ATE was affected by three-way interaction terms (see Table 2): the percentage of missingness, MNAR mechanisms, and MPA methods differentiated the bias of ATE (${PEV}_{B\times D\times E}=0.10$). The two-way interaction terms affecting bias were an interaction between the percentage of missingness and MPA methods $\left({PEV}_{B\times E}=0.11\right)$, and between MNAR mechanisms and MPA methods (${PEV}_{D\times E}=0.18$). The main effects affecting bias were the percentage of missingness $\left({PEV}_{B}=0.02\right)$, number of covariates $\left({PEV}_{C}=0.01\right)$, MNAR mechanisms $\left({PEV}_{D}=0.02\right)$, and MPA methods $\left({PEV}_{E}=0.26\right)$.

**Table 2:** Mixed-design ANOVA results

Factors	ATE		SE ATE		MSE	
Effect size	${\upeta }_{\mathrm{G}}^{2}$	*PEV*	${\upeta }_{\mathrm{G}}^{2}$	*PEV*	${\upeta }_{\mathrm{G}}^{2}$	*PEV*
Sample size (A)	0.00	0.00	0.00	0.00	0.02	0.19
Percentage of missingness (B)	0.00	0.02	0.00	0.03	0.00	0.02
Number of covariates (C)	0.00	0.01	0.00	0.00	0.02	0.22
Missing mechanisms (D)	0.00	0.02	0.00	0.00	0.00	0.00
MPA methods (E)	0.00	0.26	0.16	0.63	0.02	0.21
(B)$\times$(E)	-	0.11	0.04	0.13	-	0.04
(D)$\times$(E)	-	0.18	-	-	-	-
(B)$\times$(D)	-	-	-	-	-	-
(C)$\times$(E)	-	-	0.03	0.08	0.02	0.16
(A)$\times$(B)$\times$(E)	-	-	-	**0.02**	-	**0.02**
(A)$\times$(C)$\times$(E)	-	-	-	**-**	-	**0.02**
(B)$\times$(C)$\times$(E)	-	-	-	**0.02**	-	**0.02**
(B)$\times$(D)$\times$(E)	-	**0.10**	-	-	-	-

*Note*. Report ${\eta }_{G}^{2}\ge 0.01$ or *PEV* $\ge$ 0.01

**Fig. 1:**
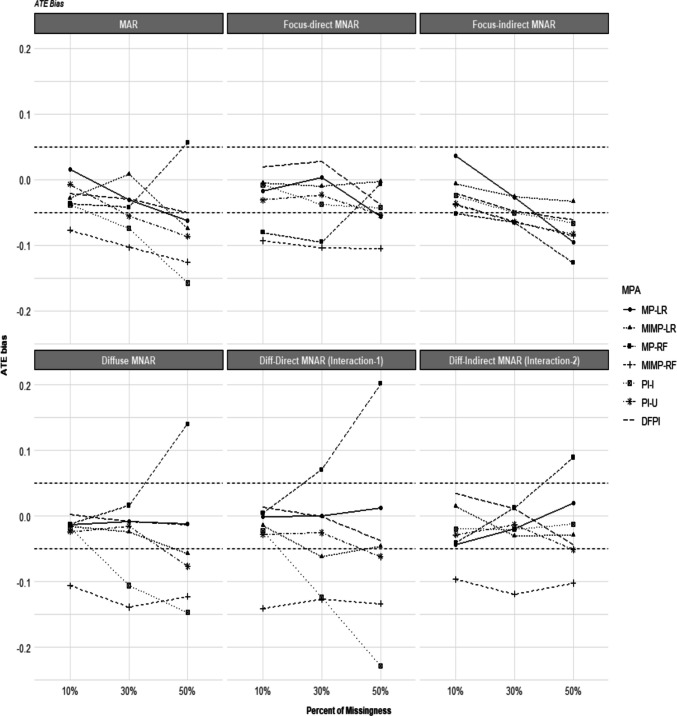
Relative bias of ATE estimates by MNAR mechanisms, percentage of missingness per MPA methods

We found that the DFPI met the acceptable bias criterion for the ATE in nearly all conditions, including the most challenging settings with 50% missingness in FI-MNAR mechanisms. In contrast, MP-LR and MIMP-LR showed unacceptable bias primarily when missingness exceeded 30% under FD, FI-MNAR, and DF-MNAR-INT2 (MP-LR method), and DF-MNAR and DF-MNAR-INT1 (MIMP-LR method). The PI-U method also performed competitively, but its bias was larger than DFPI’s in high-missingness conditions (see Table 3 and Fig. 1). The ATE estimates from the MP-RF and MIMP-RF methods had acceptable bias only in the 10% or 30% of missingness in MAR, FI-MNAR, and DF-MNAR conditions, and the interaction in two DF forms of MNAR. The general PI-I approach had inconsistent performance, showing an acceptable bias of ATE in FD, FI-MNAR, and DF-MNAR-INT2 mechanisms.

**Table 3:** Relative bias of ATE estimates by MNAR mechanisms, percentage of missingness per MPA methods

Mechanisms	Percent	MP-LR	MIMP-LR	MP-RF	MIMP-RF	PI-I	PI-U	DFPI
MAR	10%	0.015	−0.028	−0.037	−0.077	−0.038	−0.007	−0.021
	30%	−0.030	0.008	−0.042	**−0.103**	**−0.074**	**−0.056**	−0.030
	50%	**−0.063**	**−0.075**	**0.056**	**−0.125**	**−0.158**	**−0.086**	−0.048
FD-MNAR	10%	−0.018	−0.006	**−0.080**	**−0.093**	−0.008	−0.031	0.020
	30%	0.004	−0.010	**−0.095**	**−0.103**	−0.038	−0.023	0.028
	50%	**−0.056**	−0.022	**−0.066**	**−0.105**	−0.043	**−0.054**	−0.038
FI-MNAR	10%	0.036	−0.007	**−0.052**	−0.038	−0.024	−0.037	−0.021
	30%	−0.026	−0.025	**−0.065**	**−0.064**	**−0.050**	**−0.064**	−0.047
	50%	**−0.095**	−0.033	**−0.127**	**−0.085**	**−0.067**	**−0.083**	**−0.062**
DF-MNAR	10%	−0.014	−0.016	−0.013	**−0.106**	−0.018	−0.024	0.002
	30%	−0.008	−0.024	0.016	**−0.139**	**−0.106**	−0.016	−0.009
	50%	−0.012	**−0.057**	**0.140**	**−0.123**	**−0.147**	**−0.077**	−0.013
DF-MNAR-INT1	10%	−0.001	−0.015	0.004	**−0.141**	−0.023	−0.028	0.014
	30%	0.000	**−0.062**	**0.070**	**−0.127**	**−0.124**	−0.026	−0.001
	50%	0.012	**−0.064**	**0.202**	**−0.134**	**−0.229**	**−0.062**	−0.038
DF-MNAR-INT2	10%	−0.044	0.015	−0.041	**−0.097**	−0.020	−0.029	0.035
	30%	**−0.091**	−0.030	0.012	**−0.119**	−0.020	−0.013	0.011
	50%	**0.093**	−0.029	**0.089**	**−0.103**	−0.012	**−0.051**	−0.044

Bold: $$\left|\widehat{Bias}\left(ATE\right)\right|$$ $$\ge$$ 0.05

### Relative bias of the SE of ATE

The effect sizes from the ANOVA in Table 2 on the relative bias of the standard error of the ATE indicate that it was affected by two three-way interaction terms consisting of the sample size, percentage of missingness, and MPA methods $\left({PEV}_{A\times B\times E}=0.02\right)$, and percentage of missingness, number of covariates, and MPA methods $\left({PEV}_{B\times C\times E}=0.02\right)$. With the MPA methods, the two-way interaction terms with percentage of missingness $\left({PEV}_{B\times E}=0.13\right)$ or number of covariates $\left({PEV}_{C\times E}=0.08\right)$ provided large effect size. As a main effect, the percentage of missingness (${PEV}_{B}=0.03$) and MPA methods $\left({PEV}_{E}=0.63\right)$ differentiated the bias of SE of ATE.

Regarding the standard error of the ATE, DFPI and PI-U yielded acceptable bias across almost all combinations of sample size, number of covariates, and percentage of missingness, whereas MP-based methods (e.g., MP-LR and MP-RF) exhibited systematically underestimated standard errors (see Tables 4 and 5). A MIMP-RF method had an acceptable bias of standard error of ATE regardless of the percentage of missingness with both 500 and 2,000 sample sizes. With a small sample size, the PI-I method was acceptable only at a small portion of missingness, but it became an acceptable bias of SE ATE in both 10% and 30% missingness as sample size increased.

**Table 4:** Relative bias of the standard error of ATE by sample size, percentage of missingness per MPA methods

	MP-LR	MIMP-LR	MP-RF	MIMP-RF	PI-I	PI-U	DFPI
500							
10%	**−0.169**	0.027	**−0.124**	0.097	−0.032	0.000	0.004
30%	**−0.210**	**0.184**	**−0.155**	0.088	**−0.147**	−0.005	−0.013
50%	**−0.224**	**0.409**	**−0.207**	0.053	**−0.283**	−0.035	−0.071
2,000							
10%	**−0.180**	0.079	**−0.121**	0.096	0.016	0.056	0.034
30%	**−0.225**	**0.128**	**−0.170**	0.031	−0.059	0.019	−0.058
50%	**−0.268**	**0.171**	**−0.184**	−0.017	**−0.196**	−0.067	−0.092

Bold: $$\left|\widehat{Bias}(SE\left(ATE\right))\right|$$ $$\ge$$ 0.1

**Table 5:** Relative bias of standard error of ATE by number of covariates, percentage of missingness per MPA methods

	MP-LR	MIMP-LR	MP-RF	MIMP-RF	PI-I	PI-U	DFPI
10							
10%	−0.069	0.057	**−0.132**	**0.146**	−0.014	0.022	0.019
30%	**−0.121**	**0.120**	**−0.162**	**0.119**	**−0.100**	0.000	−0.055
50%	**−0.172**	**0.177**	**−0.189**	**0.175**	**−0.216**	−0.055	**−0.159**
20							
10%	**−0.280**	0.048	**−0.112**	0.046	−0.002	0.034	0.019
30%	**−0.315**	**0.193**	**−0.164**	0.000	**−0.105**	0.014	−0.016
50%	**−0.320**	**0.402**	**−0.202**	−0.039	**−0.263**	−0.046	**−0.104**

Bold: $$\left|\widehat{Bias}(SE\left(ATE\right))\right|$$ $$\ge$$ 0.1

### Mean squared error (MSE)

Regarding the MSE, Table 2 indicates that it was affected by three three-way interactions of (1) sample size, percentage of missingness, and MPA methods $\left({PEV}_{A\times B\times E}=0.02\right)$, (2) sample size, number of covariates, and MPA methods $\left({PEV}_{A\times C\times E}=0.02\right)$, and (3) percentage of missingness, number of covariates, and MPA methods $\left({PEV}_{B\times C\times E}=0.02\right)$. With the MPA methods, the two-way interaction terms were shown with percentage of missingness (${PEV}_{B\times E}=0.04$) or number of covariates $\left({PEV}_{C\times E}=0.16\right)$ as a large effect size. As a main effect, sample size $\left({PEV}_{A}=0.19\right)$, the percentage of missingness $\left({PEV}_{B}=0.02\right)$, number of covariates $\left({PEV}_{B}=0.22\right)$, and MPA methods $\left({PEV}_{E}=0.21\right)$ differentiated the MSE. Across the three MSE tables (Tables 6, 7, and 8), overall accuracy improved with larger sample size and fewer covariates, and proximity-based approaches (PI-U and DFPI) were consistently among the best-performing methods. In Table 6, the MSE was substantially smaller at a sample size of 2,000 than a sample size of 500 for all methods. At a sample size of 500, MIMP-RF yielded the lowest MSE across percentage of missingness, with DFPI and PI-U close behind. At a sample size of 2,000, the MSE for PI-U and DFPI remained very low and slightly better than for MIMP-LR and MIMP-RF, while MP-LR and MP-RF remained higher. Table 7 shows that increasing the number of covariates drastically increased the MSE for MP-LR and PI-I, while MIMP-RF was the most robust and PI-U and DFPI stayed comparatively low. Finally, Table 8 reinforces these patterns: DFPI achieved the lowest MSE under the simpler setting (500 sample, 10 covariates), but MIMP-RF tended to be best under a small sample size and high-dimensional setting (sample size of 500, 20 covariates). For the sample size of 2,000, although MIMP-LR was best at the 10-covariate and PI-U was best at the 20-covariate setting, DFPI remained among the top performers across both covariate conditions.

**Table 6:** Mean squared error (MSE) of ATE by sample size, percentage of missingness per MPA methods

	MP-LR	MIMP-LR	MP-RF	MIMP-RF	PI-I	PI-U	DFPI
500							
10%	0.748	0.374	0.382	0.245	0.336	0.286	0.282
30%	0.941	0.391	0.453	0.239	0.564	0.277	0.263
50%	0.901	0.471	0.622	0.260	1.126	0.284	0.275
2,000							
10%	0.389	0.066	0.134	0.069	0.071	0.060	0.061
30%	0.459	0.071	0.170	0.074	0.107	0.063	0.065
50%	0.447	0.084	0.229	0.084	0.240	0.073	0.082

**Table 7:** Mean squared error (MSE) of ATE by number of covariates, percentage of missingness per MPA methods

	MP-LR	MIMP-LR	MP-RF	MIMP-RF	PI-I	PI-U	DFPI
10							
10%	0.132	0.095	0.195	0.095	0.102	0.089	0.084
30%	0.146	0.102	0.226	0.089	0.150	0.092	0.085
50%	0.148	0.122	0.303	0.095	0.275	0.101	0.097
20							
10%	1.004	0.345	0.321	0.218	0.305	0.257	0.259
30%	1.254	0.360	0.397	0.223	0.520	0.248	0.243
50%	1.200	0.432	0.548	0.248	1.091	0.255	0.260

**Table 8:** Mean squared error (MSE) of ATE by sample size, number of covariates per MPA methods

	MP-LR	MIMP-LR	MP-RF	MIMP-RF	PI-I	PI-U	DFPI
500							
10	0.212	0.175	0.357	0.146	0.284	0.148	0.136
20	1.515	0.649	0.614	0.350	1.067	0.417	0.410
2,000							
10	0.072	0.037	0.126	0.041	0.067	0.040	0.041
20	0.791	0.110	0.230	0.110	0.211	0.090	0.098

### Illustrative example

The objective of the illustrative example is to demonstrate the performance of missing data imputation methods for PSA under the MNAR mechanism. We estimated the effect of mothers having a job that provides or subsidizes childcare on the duration of mothers’ breastfeeding. This is an extension of an example provided in Leite (2017), which was motivated by Jacknowitz (2008). The example uses data from the National Longitudinal Survey of Youth 1979 (NLSY79) and NLSY79 Child and Young Adult.^5^ Notably, the NLSY79 dataset includes many sensitive questions such as the class of workers at current job, earning wages, and frequency of arguments with husband about chores and responsibilities. We assumed that these sensitive questions may lead to missing responses based on MNAR mechanisms (Enders, 2010; van Buuren, 2018).

#### Participants

The NLSY79 sample for an illustrative example includes 1,209 children and their mothers (Leite, 2017; Leite et al., 2021). Due to the availability of the treatment variable of interest, we restricted the dataset to NLSY79 waves from 1988 to 1994, 1996, 1998, 2000, 2002, 2004, 2006, 2008, and 2010. The sample was restricted to mothers who had a child and had worked at least one job in the fourth quarter of pregnancy and returned to the workplace within 12 weeks of the child's birth. Considering the oversampling process used in the NLSY79 dataset, it is hard to say that sampled working mothers represent the population of working mothers. For this reason, we did not use sampling weights to analyze this dataset.

#### Measures

The treatment variable is whether the mother’s employer provided or subsidized childcare (1 = employment, 0 = non-employment). As a summary, 107 (8.85%) mothers in the sample were employed, and 1,102 (91.5%) mothers were not. There was no missingness in the treatment variable, but the outcome variable (age in weeks of the child when mothers stopped breastfeeding) had 163 cases (13.48%) with missing values. The outcome variable follows a right-skewed distribution, indicating that most mothers stopped breastfeeding early. The covariates used in the PSA were assumed to have relationships with mothers having a job that provides or subsidizes childcare and the duration of the mother’s breastfeeding. Based on theoretical relationships and statistical grounds (Jacknowitz, 2008; Leite, 2017; Leite et al., 2021), a total of 31 covariates were used in this analysis. Across all covariates, 16.9% missing data is reported, and each covariate has at least 5% of missingness. The highest portion of missingness is for the question about the frequency of arguments with the husband about chores and responsibility (781 cases: 64.59%). Among the covariates, 12 categorical covariates were transformed into dummy-coded variables. Also, the continuous variables (19 covariates) with right-skewed distributions were log-transformed.

#### Analysis

The analysis followed these steps:

1. Generate the missing indicators for 31 covariates and group the missingness patterns with at least 100 observations per group;
2. Perform the six types of MPA that use the random forest and estimate the PS in the NLSY79 dataset;
3. Evaluate the overlap of estimated PS of the treated and untreated groups;
4. Obtain the inverse probability of treatment weights (IPTW) through PS for the average treatment effect on the treated (ATT);
5. Evaluate the covariate balance through IPTW; and
6. Estimate the ATT.

In the first step, missing indicators were generated based on the missing information for the 31 covariates and missingness patterns were grouped following the recommendation of Qu and Lipkovich (2009) for the MP-LR and MP-RF methods. The generated missing indicators were included either as covariates in MIMP or as imputation processes in PI-based methods (PI-I, PI-U, DFPI).

In the second step, six types of MPA methods were applied to the NLSY79 dataset. MP-LR and MP-RF estimated the PS directly on each missingness pattern, using either logistic regression or random forest. These PS models were fitted for each missingness pattern after initially inputting the average value of non-missing observations in other patterns (Qu & Lipkovich, 2009). MIMP-LR and MIMP-RF followed the same principle as the MIMP method, so that PS was estimated with missing indicators after imputing missing data through MI. As a pre-imputation process, except for the MIMP-LR and MIMP-RF, PI-I implemented proximity imputation with missing indicators. These methods estimated PS without missing indicators in the PS model. They were implemented through the *mice* (van Buuren & Groothuis-Oudshoorn, 2011), *randomForest* (Liaw & Wiener, 2002), and *missForest* (Stekhoven & Bühlmann, 2012) packages in R software. If a method used the MICE algorithm, we generated 10 imputed datasets. For all RF methods, 500 trees were used. For estimating the PS, either logistic regression or RF with 31 covariates, with or without missing indicators, were fitted to the dataset by using the *glm or randomForest* functions in R (Liaw & Wiener, 2002).

In the third step, we evaluated common support between the treated and control groups by checking the distribution of PS values. Poor common support may result in extreme values of PS weights (Rosenbaum & Rubin, 1983; Stuart, 2010). In the fourth step, we estimated PS weights for estimating the ATT (Leite, 2017):

23 $${w}_{i}={Z}_{i}+\frac{\left(1-{Z}_{i}\right){e}_{i}\left(X\right)}{1-{e}_{i}\left(X\right)}$$

where

${Z}_{i}$: is the dummy treatment indicator, and
${e}_{i}\left(X\right)$: *is the estimated PS.*

This PS weight was calculated using the 10 imputed datasets for MIMP-LR and MIMP-RF, and a single imputed dataset for PI-I methods.

In the fifth step, we evaluated covariate balance by comparing the PS weighted means of the covariates between the treatment and control groups. We used the baseline equivalence criteria of the Procedures and Standards Handbook of the What Works Clearinghouse (WWC; U.S. Department of Education, Institute of Education Sciences, & What Works Clearinghouse, 2017): covariates with a standardized difference between the treated and control groups smaller than 0.05 were regarded as balanced. For the 10 imputed datasets, when calculating the standardized differences, weighted mean differences (or proportions) and their standard deviation, which were taken across 10 imputed datasets, were aggregated by taking the mean (Leite et al., 2021).

In the sixth step, the ATT was estimated using a Poisson regression model

$$\mathrm{log}({Y}_{i})={\gamma }_{0}+{\gamma }_{1}{Z}_{i}$$

where

${Y}_{i}$: is the age in weeks of the child when the mother stopped breastfeeding,
${Z}_{i}$: is the working status of mothers for the job that provides or subsidizes childcare (treatment),
${\gamma }_{0}$: is the intercept of the log of duration of breastfeeding for unemployed mothers, and
${\gamma }_{1}$: is the ATT. The standard error of the ATT was obtained by Taylor series linearization using the *survey* package in R (Heeringa et al., 2010; Lumley, 2004).

Using the 10 imputed datasets, an ATT estimate and its standard error were calculated following Rubin’s rule (Rubin, 1987).

## Results

### Common support

**Fig. 2:**
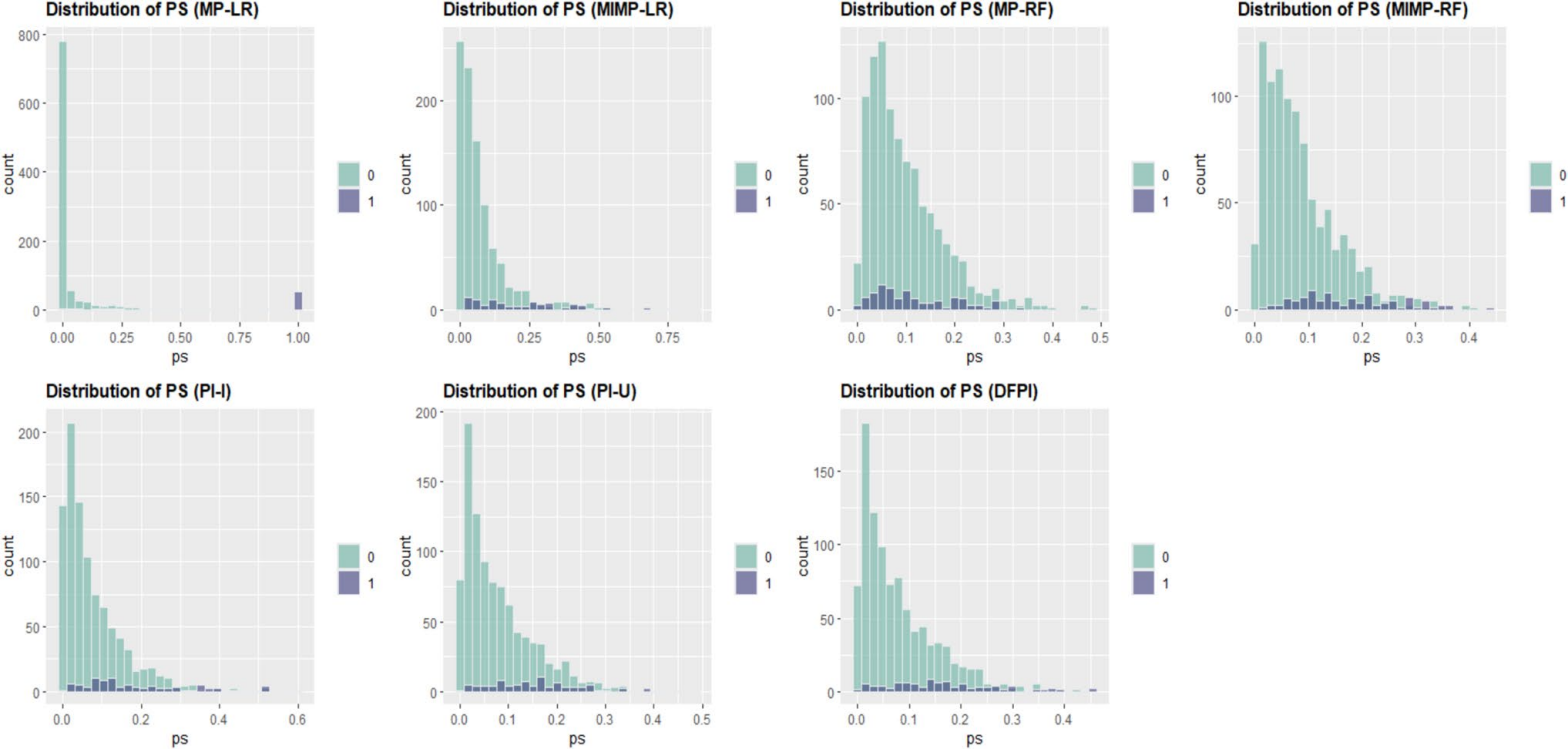
Overlapping pattern in the distribution of PS per missingness pattern methods

The common support of PS in almost all types of missingness pattern methods offered an adequate distributional overlapping pattern, usually shown in the ATT setting. The area of distribution of PS in the control group covered that of the treated group. However, the MP-LR method presented a distribution that was highly skewed for the group, resulting in poor common support (Fig. 2).

### Covariate balance

Table 9 shows the absolute standardized effect sizes of covariates for data with eight types of missingness pattern methods after implementing PSA.^6^ MIMP-LR has the smallest maximum absolute standardized effect size and number of unbalanced covariates, meeting the criterion for the WWC (mean = 0.022; max = 0.111; 14 covariates exceeding 0.05). Among the RF-based pre-imputation approaches, PI-I also performed well in terms of balance (mean = 0.036; max = 0.195; 19 covariates exceeding 0.05), and the PI-U and DFPI methods showed similarly favorable balance (means $\approx$ 0.030; 15–16 covariates exceeding 0.05). In contrast, we found that the MP-based methods, including MP-LR and MP-RF, had the largest number of unbalanced covariates, exceeding the 0.05 criterion (i.e., 51–59 covariates exceeding 0.05). The specific standarized mean differences of covariates across methods is presented in Appendix A.

**Table 9:** Summary of absolute standardized effect sizes of covariates per missingness pattern (MP) methods in the illustrative example

Imputation methods	Minimum	Mean	Maximum	Number of covariates exceeding 0.05 *SD*
MP-LR	0.000	0.107	0.433	59
MIMP-LR	0.000	0.022	0.111	14
MP-RF	0.000	0.105	0.640	51
MIMP-RF	0.000	0.097	0.537	52
PI-I	0.000	0.036	0.195	19
PI-U	0.000	0.030	0.265	15
DFPI	0.000	0.029	0.253	16

### Treatment effect

The estimated ATTs are shown in Table 10. None were statistically significant, indicating that there was no effect of working for a company that provides childcare on the child’s age when the mother stopped breastfeeding. This result is similar to that of Leite (2017) and Leite et al. (2021). The standard errors of ATT were almost identical, except for the largest standard errors resulting from the MP-LR method.

**Table 10:** ATT, SE, and *p*-values per imputation method in the illustrative example

Imputation methods	Estimates (${\gamma}_{1}$)	*SE* (${\gamma}_{1}$)	*p*-value
MP-LR	0.173	0.308	0.574
MIMP-LR	0.233	0.208	0.264
MP-RF	0.299	0.187	0.11
MIMP-RF	0.241	0.188	0.201
PI-I	0.283	0.189	0.134
PI-U	0.297	0.188	0.116
DFPI	0.306	0.188	0.106

## Discussion of the illustrative example

The illustrative example demonstrates how the proposed missingness pattern approaches (MPA) can be implemented in an applied setting and how results can be interpreted alongside practical diagnostics such as overlap and covariate balance. As shown in Fig. 2, most approaches yielded an adequate overlap in propensity score distributions, suggesting that the common support requirement was broadly satisfied in this dataset. However, the approaches differed in post-weighting balance, highlighting the value of routinely examining balance diagnostics rather than relying solely on a single treatment effect estimate.

In terms of covariate balance (see Table 9), MIMP-LR produced the best covariate balance. Importantly, in practice, PI-I also achieved relatively strong balance, and PI-U and DFPI performed similarly well. In contrast, MP-based approaches left substantially more imbalanced covariates. Therefore, this example shows that methods incorporating either multiple imputation (e.g., MIMP-LR) or proximity-based pre-imputation (e.g., PI-I, PI-U, DFPI) can lead to more favorable balance and potentially more stable propensity score weights than direct missingness pattern propensity score estimation. At the same time, balance should be interpreted cautiously in the present context: good balance on observed and imputed covariates does not necessarily eliminate the bias of treatment effects if the imputation model does not recover the relevant unobserved confounding structure. Nevertheless, the balance observed for PI-I and MIMP-LR suggests that they can be useful options in practice, while our example provides complementary evidence of when more robust methods (e.g., DFPI and PI-U) may be preferable under severe or complex MNAR mechanisms.

Despite these covariate balance diagnostic differences, the estimated ATT was consistent across methods (Table 10). The ATT estimates ranged from 0.173 (MP-LR) to 0.306 (DFPI) and none of the *p*-values were below 0.05, indicating that the substantive conclusion was not sensitive to the choice among these approaches in this illustration. At the same time, MP-LR produced the largest standard error (*SE* = 0.308), whereas the other approaches yielded similar SEs (approximately 0.187–0.208), which is consistent with the observation that differences in overlap and balance may translate into differences in estimator stability. Because the missing data mechanism in an applied dataset cannot be verified, these results should be interpreted as illustrative. Nevertheless, they underscore a practical workflow in which analysts compare multiple approaches and place particular emphasis on overlap and balance diagnostics when selecting or reporting PSA results.

## Discussion and conclusion

The Monte Carlo simulation suggested that DFPI (and, in many conditions, PI-U) tended to yield comparatively low bias and MSE under the MAR and MNAR mechanisms in PSA. However, their relative performance varied across missingness mechanisms and severity. In particular, DFPI achieved acceptable ATE bias with nearly all mechanism-by-missingness combinations, but we also observed settings in which DFPI underperformed in certain conditions (e.g., the FI-MNAR mechanism at 50% missingness) as compared to the other methods (i.e., PI-U, PI-I, and MIMP-LR). This finding indicates that the relative advantages of RF and LR-based methods are context-dependent. Therefore, we can conclude that DFPI is a promising and generally robust approach rather than a universally dominant solution. The strengths of DFPI and PI-U that we identified are supported by earlier findings of Tang and Ishwaran (2017) and Beesley and Taylor (2021), who showed that the RF-based imputation remains stable even under the non-monotonic and complex missing data mechanisms. Although the distance metric employed in PI-I, PI-U, and DFPI is not identical to the distance-based, semi-supervised kernel method proposed by Lan et al. (2021), all three methods were RF-based proximity-driven and demonstrated comparable robustness in high-missingness settings. Therefore, our results can be partially supported by Lan et al. (2021), who reported strong performance of their method despite the high rate of MNAR missingness. Even though PI-U, PI-I, and DFPI often performed similarly as these rely on RF-derived proximity structures for pre-imputation of missing confounders, DFPI is the only method that combines matrices from treatment-focused and outcome-focused forests. Its dual-forest construction is expected to yield the clearest incremental benefit when missingness pattern interacts with both treatment assignment and outcome processes (e.g., in more complex, nonlinear, or interaction-driven MNAR settings).

Our simulation results show that the MP and MIMP methods using logistic regression (i.e., MP-LR and MIMP-LR) generally produced less biased ATE estimates than those using random forests (i.e., MP-RF and MIMP-RF). However, when the percentage of missingness was high (i.e., 50%), MP-LR provided unacceptable bias in ATE in the FD-MNAR and FI-MNAR mechanisms, whereas indicator-augmented MIMP-LR still had an acceptable bias in those cases. This suggests that when the missingness in confounders follows this MNAR mechanism and the missingness rate is high, adding missing indicators in the PS model is crucial for reducing bias. However, when the probability of missingness depended on both the missing and observed values of covariates, such as the DF-MNAR mechanisms, even MP-LR produced an acceptable level of the bias of ATE. Additionally, we found that substituting logistic regression with a random forest in the MP and MIMP methods led to worse performance compared to the LR counterparts with respect to the bias of ATE. One possible explanation is that, in pattern-stratified implementations (e.g., MP-RF/MIMP-RF), fitting RF models within pooled missingness-pattern strata can reduce the effective sample size per fit and increase variability in propensity score estimation under high missingness. This reflects a bias–variance trade-off and motivates careful tuning and alternative RF-based strategies rather than indicating a general limitation of random forests (Kirasich et al., 2018). We can also explain the reason for the relatively strong performance of MP-LR (or MIMP-LR), due to the LR-based simulation setting that tends to work well in LR-based propensity score estimation. The treatment assignment in the data-generating process followed a logistic regression, and thus the MP-LR (or MIMP-LR) closely approximates the true propensity score surface when missingness is moderate and missingness pattern strata remain sufficiently large.

Consistent with prior studies, the simulation shows that MP-LR (Qu & Lipkovich, 2009) and MIMP-LR (Qu & Lipkovich, 2009; Choi et al., 2019; Leite et al., 2021) tend to have acceptable SE ATE bias, with a small percentage of missingness and few covariates with missing values. This may be because conditions with a small amount of missingness on a few covariates are likely to result in the same missingness pattern. In such situations, the missingness pattern groups are relatively homogeneous, which leads to unbiased estimation of ATE and its SE under an MNAR mechanism.

In this study, simulation and an illustrative example served different purposes with respect to uncertainty quantification. The simulation evaluated whether estimated SEs were calibrated to the empirical SE defined by repeated sampling. Thus, the operational definition of “acceptable SE ATE bias” used in this study should be understood as good calibration to the across-replication empirical SE under those low-missingness, homogeneous-pattern conditions. In contrast, the illustrative example resulted in a model-based SE estimate in a single dataset, and thus is more sensitive to application-specific features such as overlap, extreme PS weights, and finite-sample instability. Therefore, even when MP-LR shows acceptable SE bias in the simulation under small missingness, its single sample SE in the illustrative example can still be comparatively large if the empirical setting exhibits poor common support and unstable PS weights. Making this distinction explicit helps reconcile the negative SE bias patterns for MP-LR in the simulation with its comparatively large single-sample SE in the illustrative example.

In the present study, four main limitations should be noted: First, we did not test prediction-based RF imputation methods that incorporate missing indicators, such as the *missForest* algorithm proposed by Stekhoven and Bühlmann (2012). According to previous studies, including missing indicators in the imputation model decreases bias in the estimates of causal effect, as these indicators serve as proxies for unmeasured variables (Sperrin & Martin, 2020; Beesley & Taylor, 2021). Using the *missForest* algorithm with missing indicators might impute values that can capture not only the missingness pattern but also the interactions and nonlinear relationships between observed variables and missing indicators. Second, the simulation approach could not limit the occurrence of extreme PS, which violates the positivity assumption. Although the simulation in this study followed Qu and Lipkovich’s (2009) recommendation of using 100 cases per missingness pattern for stable estimation of ATE, we observed that 10% to 40% of simulated datasets for the MP and MIMP methods still produced extreme PS close to 0 or 1 in some conditions. This problem was more pronounced for the MP method (e.g., MP-RF) than the MIMP method. MP-RF showed a higher error rate in estimating PS compared to MP-LR, indicating that it struggled with extreme PS. Third, the choice of hyperparameter in RF can influence the resulting imputations (e.g., PI-I, PI-U, DFPI), PS estimation (e.g., MP-RF, MIMP-RF), and downstream treatment effect estimates. Hyperparameters that control tree complexity and locality (e.g., minimum node size or maximum depth) affect whether proximities emphasize very local neighborhoods versus smoother, more global similarity (Lin & Jeon, 2006; Wager & Athey, 2018). Hyperparameters that control randomness and feature exploration (e.g., the number of candidate variables per split, *mtry*) can change which covariate interactions are captured and therefore which units are considered “similar” by the induced forest weights (Breiman, 2001; Probst et al., 2019). Finally, the number of trees primarily affects Monte Carlo variability: because proximities are computed by aggregating co-occurrences in terminal nodes across trees, using too few trees can yield noisier proximity estimates and less stable propensity score predictions, which may propagate into more variable propensity scores with potentially more variable IPTW weight (Breiman, 2001; Austin & Stuart, 2015). In this study, we used a single, prespecified set of hyperparameters across RF-based approaches to ensure that differences in performance were attributable to the methodological differences (e.g., single unsupervised proximity vs. dual-forest proximity) rather than to method-specific tuning. We acknowledge that alternative hyperparameter tuning could shift the bias–variance trade-off and may improve performance in particular settings; developing principled tuning strategies for RF-based missing data methods remains an important direction for future research. Fourth, even though treatment indicator and outcome may also be partially observed and can plausibly follow the MNAR mechanism, we focused on the MNAR mechanism in the baseline confounders. Handling missing values in treatment and outcome variables typically requires additional assumptions (Howe et al., 2016; Zhang et al., 2016; Mitra, 2023) and is often studied as a separate issue (Penning de Vries & Groenwold, 2018; Leyrat et al., 2019; Blake et al., 2020) from missing confounders in PSA. Extending DFPI to settings with MNAR outcome missingness is particularly relevant because DFPI’s second step involves outcome prediction; future work should investigate DFPI under joint missingness in confounders and outcomes and evaluate how combining processes in DFPI affect bias and uncertainty quantification.

Beyond this limitation, two broader research directions deserve attention. First, several emerging methods for MNAR mechanisms can be combined into PSA. For example, Beesly and Taylor (2021) proposed a weighted analysis on stacked multiple imputations; Haliduola et al. (2022) used an oversampling method after *k*-means clustering followed by a recurrent deep neural network for imputing missing values; and Chen et al. (2022) used pseudo-likelihood estimation based on a synthetic distribution that involves unknown PS. These three methods, like the PI and DFPI methods in our research, commonly implement missingness patterns through advanced machine learning and neural network techniques. For future studies, therefore, it would be worth comparing these new methods with the PI or DFPI methods under the various MNAR mechanisms.

Research is still needed regarding the pooling process used in the missingness pattern methods. This study followed Qu and Lipkovich’s (2009) pooling process, which utilizes Euclidean distance to group similar missingness patterns and prioritize merging the largest pattern groups (up to 100 cases). As this process is affected by the sample size, proportion of missingness, and number of covariates, the robustness of estimation using the MP method needs to be evaluated compared with other pooling strategies (e.g., unsupervised clustering methods like *k*-means or hierarchical clustering). Moreover, instead of depending solely on the Euclidean distance of each missingness pattern, one could consider the position of occurrence of missingness within a response of an individual (e.g., which specific survey items are missing) in the pooling process. This is helpful for capturing the similarity of missing responses in the context of social science surveys and psychometric scales.

In conclusion, this study introduced several RF-based extensions to existing methods (MP-RF, MIMP-RF, PI-I, PI-U, DFPI) and compared them with LR-based methods for MNAR data in PSA. Across simulation conditions, DFPI emerged as a promising and generally robust proximity-based imputation approach when key confounders were missing under MAR and a range of MNAR mechanisms. DFPI tended to yield consistently small bias in ATE and its estimated standard error, although it was not uniformly superior across all individual conditions. Moreover, PI-U showed comparable performance in many scenarios, and MP/MIMP-LR methods performed well when their assumptions were approximately met and missingness was not extreme.

For applied researchers, our results can provide several practical decision criteria when choosing among these approaches. First, when the percentage of missingness is relatively small and missingness pattern groups are sufficiently large and homogeneous, MP-LR or MIMP-LR may provide a simple and effective option. Second, when missingness is moderate-to-high, missingness patterns are sparse and heterogenous, or complex nonlinearities and interactions are plausible when we use many confounders, proximity-based pre-imputation (DFPI and PI-U) may be preferable because they can better accommodate a flexible structure without relying on restrictive parametric specifications. Third, regardless of the chosen method, researchers should routinely examine overlap and post-weighting covariate balance. If severe instability of imbalance persists, changing methods, refining tuning parameters in using RF, or applying stabilization strategies should be considered. Taken together, we view DFPI as a useful addition to the analyst’s toolkit for PSA with MNAR covariates rather than as a universal replacement for existing approaches.

## Supplementary Information

Below is the link to the electronic supplementary material.

Supplementary file1 (DOCX 716 KB)

## Data Availability

The data used in the illustrative example are from the National Longitudinal Survey of Youth 1979 (NLSY79) dataset, which was conducted by the U.S. Bureau of Labor Statistics. A public version of the NLSY79 data can be accessed at https://www.nlsinfo.org/content/access-data-investigator/accessing-data-cohorts.

## Footnotes

1 As more imputed datasets are generated, the pooled ATE estimates become less affected by within-variability of ATE estimates from these imputed datasets. Hence, the results from 10 imputed datasets are typically more reliable than those from five imputed datasets commonly used by many researchers (Enders, 2010).

2 All R scripts for generating the simulated data, fitting the MPA models, and evaluating the results are archived in the “simulation” directory of the supplementary materials on the Open Science Framework (OSF) (https://osf.io/7y25h/?view_only=03473b01e272410b81c24ef6cceafe71).

3 The population correlation matrix derived from the NLSY79 dataset is saved as an R file (“sim_correlation_matrix.RData”) in the simulation folder on the Open Science Framework (OSF) (https://osf.io/7y25h/?view_only=03473b01e272410b81c24ef6cceafe71).

4 The **mice** function in the *mice* package specifies the imputation model using the method option in the code. For the MIMP-LR and MIMP-RF, this study used “Predictive Mean Matching (PMM)” as the imputation model.

5 Analysis code is available at the “Illustrative Example” directory of the supplementary materials on the Open Science Framework (OSF) (https://osf.io/7y25h/?view_only=03473b01e272410b81c24ef6cceafe71). The illustrative example uses data from the publicly accessible NLSY79 dataset (https://www.bls.gov/nls/), which is not hosted in the repository. Instructions for accessing the data are provided in the analysis code. This study was not preregistered.

6 Standardized effect sizes of all covariates and their missing indicators per imputation methods are shown in Appendix A

## Code Availability

All supplemental materials including R scripts of simulation and illustrative example analysis, are provided via the Open Science Framework (OSF) at https://osf.io/7y25h/?view_only=03473b01e272410b81c24ef6cceafe71.
