# Publisher Correction: Enhancing propensity score analysis with data missing not at random: Introducing dual-forest proximity imputation

**DOI:** 10.3758/s13428-026-03094-x

**Published:** 2026-06-08

**Authors:** Yongseok Lee, Walter L. Leite

**Affiliations:** 1 Department of Human Development and Family Science, Purdue University, Hanley Hall (Room 356), 1202 Mitch Daniels Blvd, West Lafayette, IN 47906 USA; 2 School of Human Development and Organizational Studies in Education, University of Florida, Gainesville, FL USA

**Publisher Correction: Behavior Research Methods**

10.3758/s13428-026-03024-x

The original online version of this article was revised: A citation in the Abstract, (Enders, 1979), has been removed for veracity.

In Equations 2, 4 and 5, under the heading Manipulated conditions, equations 2, 4 and 5 all begin with *logit*(*R*_*k*_). However,equations 2 and 4 incorrectly displayed *Llogit*(*R*_*k*_), while equation 5 left out the term completely. These equations have all been updated.

