# Supplementary material for: Enhancing propensity score analysis with data missing not at random: Introducing dual-forest proximity imputation
Source: Behav Res Methods. 2026 May 29;58(7):182. doi: 10.3758/s13428-026-03024-x (PMC13221353; doi:10.3758/s13428-026-03024-x)
Supplement: Supplementary file 1 — Supplementary file1 (DOCX 716 KB) [file 13428_2026_3024_MOESM1_ESM.docx]

APPENDIX A

Standardized mean difference of covariates per MPA methods

| Variable | (1) | | (2) | (3) | (4) | (5) | (6) | (7) |
| --- | --- | --- | --- | --- | --- | --- | --- | --- |
| Race:1 | | -0.079 | 0.014 | -0.004 | -0.012 | -0.030 | 0.001 | 0.002 |
| Race:2 | | 0.055 | 0.026 | 0.079 | 0.068 | 0.026 | -0.002 | -0.003 |
| Race:3 | | 0.011 | -0.036 | -0.071 | -0.054 | 0.000 | 0.001 | 0.002 |
| Gender:1 | | -0.285 | -0.014 | -0.048 | -0.036 | 0.009 | 0.000 | 0.001 |
| Gender:2 | | 0.285 | 0.014 | 0.048 | 0.036 | -0.009 | 0.000 | -0.001 |
| N of jobs Before birth | | -0.100 | 0.040 | 0.045 | 0.051 | 0.010 | -0.005 | -0.001 |
| Hours | | -0.067 | -0.036 | 0.061 | 0.045 | -0.022 | -0.026 | -0.022 |
| Earning | | -0.016 | -0.053 | -0.568 | -0.412 | -0.047 | -0.007 | -0.008 |
| Bargaining:0 | | -0.128 | 0.009 | 0.089 | 0.053 | 0.037 | 0.002 | 0.002 |
| Bargaining:1 | | 0.128 | -0.009 | -0.089 | -0.053 | -0.037 | -0.002 | -0.002 |
| Length of Gestation | | 0.051 | 0.010 | 0.077 | 0.118 | 0.007 | 0.003 | 0.000 |
| Delivered place:0 | | 0.054 | 0.008 | 0.099 | 0.112 | 0.010 | 0.010 | 0.010 |
| Delivered place:1 | | -0.054 | -0.008 | -0.099 | -0.112 | -0.010 | -0.010 | -0.010 |
| Days of Mother Stayed in Hospital | | -0.066 | -0.006 | -0.115 | -0.133 | 0.006 | -0.020 | -0.013 |
| Days of Child Stayed After delivered | | -0.152 | 0.013 | -0.076 | -0.082 | 0.000 | -0.019 | -0.016 |
| Country of Birth of Mother:1 | | -0.116 | -0.033 | -0.038 | -0.038 | -0.021 | -0.023 | -0.022 |
| Country of Birth of Mother:2 | | 0.116 | 0.033 | 0.038 | 0.038 | 0.021 | 0.023 | 0.022 |
| Qualification of Test | | 0.037 | -0.032 | -0.036 | -0.006 | -0.030 | 0.004 | 0.007 |
| Weeks after birth that mother returned to work | | 0.103 | -0.004 | -0.320 | -0.213 | -0.009 | -0.010 | -0.011 |
| Mother Age | | 0.105 | -0.066 | -0.090 | -0.112 | -0.049 | -0.014 | -0.012 |
| Class Worker:1 | | -0.157 | 0.073 | 0.121 | 0.130 | 0.022 | 0.009 | 0.007 |
| Class Worker:2 | | 0.130 | -0.050 | -0.102 | -0.125 | -0.023 | -0.009 | -0.005 |
| Class Worker:3 | | 0.134 | -0.071 | -0.011 | -0.006 | 0.003 | 0.003 | -0.002 |
| Class Worker:4 | | -0.054 | -0.010 | -0.077 | -0.023 | -0.007 | -0.007 | -0.007 |
| Class Worker:5 | | 0.000 | 0.000 | 0.000 | 0.000 | 0.000 | 0.000 | 0.000 |
| Family size | | 0.004 | -0.034 | -0.026 | -0.018 | -0.027 | -0.008 | -0.010 |
| Education | | 0.016 | -0.005 | 0.094 | 0.086 | 0.028 | 0.002 | 0.004 |
| Jobs Ever | | 0.120 | -0.062 | -0.039 | -0.036 | -0.041 | -0.019 | -0.015 |
| Marital Status:1 | | -0.019 | 0.057 | 0.076 | 0.070 | 0.054 | 0.015 | 0.013 |
| Marital Status:2 | | 0.097 | -0.070 | 0.022 | -0.024 | -0.057 | -0.020 | -0.020 |
| Marital Status:3 | | -0.125 | 0.031 | -0.135 | -0.056 | 0.015 | 0.011 | 0.012 |
| Region:1 | | 0.144 | 0.001 | -0.079 | -0.015 | -0.040 | 0.013 | 0.011 |
| Region:2 | | 0.038 | -0.010 | 0.077 | 0.074 | 0.032 | 0.012 | 0.013 |
| Region:3 | | -0.119 | 0.058 | 0.015 | 0.026 | 0.038 | -0.005 | -0.006 |
| Region:4 | | -0.039 | -0.066 | -0.030 | -0.109 | -0.047 | -0.022 | -0.020 |
| Urban:0 | | 0.034 | 0.000 | -0.216 | -0.223 | 0.005 | 0.001 | 0.001 |
| Urban:1 | | -0.034 | 0.000 | 0.241 | 0.250 | -0.005 | -0.001 | -0.001 |
| Urban:2 | | 0.000 | 0.000 | 0.000 | 0.000 | 0.000 | 0.000 | 0.000 |
| Welfare | | 0.105 | 0.111 | -0.021 | -0.022 | 0.050 | 0.059 | 0.054 |
| Work (week) | | -0.199 | -0.013 | 0.552 | 0.537 | -0.010 | 0.010 | 0.011 |
| Work (hours) | | -0.018 | -0.025 | -0.043 | -0.004 | -0.010 | -0.038 | -0.033 |
| Maternity Leave:0 | | 0.025 | -0.011 | -0.320 | -0.246 | -0.008 | -0.003 | -0.003 |
| Maternity Leave:1 | | -0.025 | 0.011 | 0.320 | 0.246 | 0.008 | 0.003 | 0.003 |
| Flexible Schedule:0 | | -0.065 | -0.007 | -0.320 | -0.259 | 0.017 | -0.012 | -0.010 |
| Flexible Schedule:1 | | 0.065 | 0.007 | 0.320 | 0.259 | -0.017 | 0.012 | 0.010 |
| Argue Chores:1 | | 0.171 | -0.018 | -0.194 | -0.091 | -0.017 | -0.012 | -0.011 |
| Argue Chores:2 | | -0.088 | 0.010 | 0.119 | -0.013 | -0.003 | -0.011 | -0.003 |
| Argue Chores:3 | | -0.184 | -0.022 | -0.079 | -0.355 | -0.004 | -0.006 | -0.017 |
| Argue Chores:4 | | 0.222 | 0.018 | 0.073 | 0.315 | 0.019 | 0.038 | 0.036 |
| Dental Insurance:0 | | -0.059 | 0.022 | -0.216 | -0.141 | 0.028 | 0.024 | 0.025 |
| Dental Insurance:1 | | 0.059 | -0.022 | 0.216 | 0.141 | -0.028 | -0.024 | -0.025 |
| Life Insurance:0 | | -0.165 | -0.039 | -0.191 | -0.135 | 0.003 | -0.005 | -0.005 |
| Life Insurance:1 | | 0.165 | 0.039 | 0.191 | 0.135 | -0.003 | 0.005 | 0.005 |
| Profit sharing:0 | | -0.004 | -0.010 | -0.044 | -0.071 | 0.022 | 0.012 | 0.014 |
| Profit sharing:1 | | 0.004 | 0.010 | 0.044 | 0.071 | -0.022 | -0.012 | -0.014 |
| Retirement:0 | | -0.048 | 0.020 | -0.130 | -0.098 | 0.034 | 0.022 | 0.021 |
| Retirement:1 | | 0.048 | -0.020 | 0.130 | 0.098 | -0.034 | -0.022 | -0.021 |
| Training:0 | | -0.090 | -0.032 | -0.640 | -0.434 | -0.004 | -0.010 | -0.011 |
| Training:1 | | 0.090 | 0.032 | 0.640 | 0.434 | 0.004 | 0.010 | 0.011 |
| Race* | | 0.000 | 0.000 | 0.000 | 0.000 | 0.000 | 0.000 | 0.000 |
| Gender* | | 0.000 | 0.000 | 0.000 | 0.000 | 0.000 | 0.000 | 0.000 |
| N of jobs* | | 0.312 | 0.018 | 0.012 | -0.057 | 0.180 | 0.126 | 0.144 |
| Hours* | | 0.312 | 0.018 | 0.012 | -0.057 | 0.180 | 0.126 | 0.144 |
| Earning* | | 0.324 | 0.033 | -0.040 | -0.118 | 0.128 | 0.049 | 0.066 |
| Bargaining* | | 0.372 | -0.052 | 0.028 | -0.017 | 0.186 | 0.106 | 0.117 |
| Length of Gestation* | | -0.098 | -0.005 | 0.078 | 0.018 | 0.147 | 0.098 | 0.098 |
| Delivered place* | | -0.140 | -0.010 | 0.067 | 0.010 | 0.127 | 0.088 | 0.089 |
| Days of Mother Stayed in Hospital* | | -0.328 | -0.009 | -0.029 | -0.153 | -0.153 | -0.106 | -0.103 |
| Days of Child Stayed After delivered* | | -0.291 | -0.009 | 0.005 | -0.115 | -0.120 | -0.075 | -0.073 |
| Country of Birth of Mother* | | 0.000 | 0.000 | 0.000 | 0.000 | 0.000 | 0.000 | 0.000 |
| Qualification of Test* | | 0.216 | 0.050 | 0.077 | 0.056 | 0.117 | 0.130 | 0.129 |
| Weeks after birth that mother returned to work* | | 0.000 | 0.000 | 0.000 | 0.000 | 0.000 | 0.000 | 0.000 |
| Mother Age* | | 0.000 | 0.000 | 0.000 | 0.000 | 0.000 | 0.000 | 0.000 |
| Class Worker* | | 0.242 | 0.021 | -0.097 | -0.202 | -0.064 | -0.265 | -0.253 |
| Family Size* | | 0.000 | 0.000 | 0.000 | 0.000 | 0.000 | 0.000 | 0.000 |
| Education* | | 0.000 | 0.000 | 0.000 | 0.000 | 0.000 | 0.000 | 0.000 |
| JobsEver* | | 0.000 | 0.000 | 0.000 | 0.000 | 0.000 | 0.000 | 0.000 |
| Marital Status* | | 0.000 | 0.000 | 0.000 | 0.000 | 0.000 | 0.000 | 0.000 |
| Region* | | 0.106 | -0.036 | 0.070 | 0.022 | 0.105 | 0.039 | 0.036 |
| Urban* | | 0.106 | -0.036 | 0.053 | 0.005 | 0.099 | 0.022 | 0.019 |
| Welfare* | | 0.000 | 0.000 | 0.000 | 0.000 | 0.000 | 0.000 | 0.000 |
| Work (weeks)* | | 0.000 | 0.000 | 0.000 | 0.000 | 0.000 | 0.000 | 0.000 |
| Work (hours)* | | 0.106 | 0.012 | -0.156 | -0.237 | -0.047 | -0.079 | -0.076 |
| MaternityLeave* | | 0.411 | -0.001 | -0.049 | -0.028 | -0.072 | -0.041 | -0.052 |
| Flexible Schedule* | | 0.245 | 0.021 | -0.083 | -0.186 | -0.056 | -0.255 | -0.243 |
| Argue Chores* | | -0.433 | 0.070 | 0.111 | 0.132 | -0.030 | 0.068 | 0.029 |
| Dental Insurance* | | 0.000 | 0.000 | 0.000 | 0.000 | 0.000 | 0.000 | 0.000 |
| Life Insurance* | | 0.106 | -0.034 | -0.012 | -0.047 | -0.014 | 0.020 | 0.022 |
| Profit Sharing* | | 0.267 | 0.008 | 0.192 | 0.108 | 0.195 | 0.177 | 0.175 |
| Retirement* | | 0.000 | 0.000 | 0.000 | 0.000 | 0.000 | 0.000 | 0.000 |
| Training* | | 0.106 | -0.011 | -0.087 | -0.110 | -0.077 | -0.149 | -0.144 |

(1): MP-LR, (2): MIMP-LR, (3): MP-RF, (4): MIMP-RF, (5): PI-I, (6): PI-U, (7): DFPI, *: Missing Indicators

APPENDIX B

Missingness Patterns Method

| Missingness Patterns (MP: Qu & Lipkovich, 2004; Blake et al., 2020) |
| --- |
| Require: $X_{ij}$ is $n\times p$ (where $i\in n,j\in p$) data matrix containing missing values, $T_{i}$ is the binary treatment indicator and $Y_{i}$ is outcome variable.  **1.** **Calculating Distance of Missing Patterns**: Each missingness pattern is represented by a vector of binary indicator, $S_{i}$, where each element $S_{ij}$ represents whether the $j^{th}$ covariate is missing ($S_{ij}=1$) or observed ($S_{ij}=0$). The distance of two missingness patterns between $i$ and $k$ observation: ($S_{i}$, $S_{k}$) is calculated based on the Euclidean distance ($d_{ij}$). $p$ is the total number of covariates.  $d_{ik}=\sqrt{\sum_{j=1}^{p} \left( S_{ij}-S_{kj} \right)^{2}}$  **2.** **Pooling Process**: For each missingness pattern $S_{i}$, count the number of observations $n_{i}$ associated with it. Then, sort all patterns $S_{1},S_{2},S_{3}\ldots,S_{C}$ in descending order based on $n_{i}$. If the number of observations for a pattern exceeds the predefined minimum threshold $n_{min}$ (e.g., 50 or 100), skip the merging process for that pattern and proceed to evaluate the next one. Otherwise, iteratively merge them with the most similar pattern (as determined by the shortest Euclidean distance) and the smallest number of observations until $n_{min}$ is achieved. If any patterns remain with insufficient sample sizes after all iterations, assign them to the group with the most similar pattern, prioritizing integration into large groups in descending order of size.  **3.** **Propensity Score Estimation**: For the dataset in each pooled cell *(*$X_{ij}^{(C)}$*,* $T_{i}^{(C)}$*,* $Y_{i}^{(C)}$)*,* fit the logistic regression model to estimate the propensity score: $\hat{p}_{i}^{(C)}$, which includes all the variables that are non-missing in any of the patterns within the pooled cell. For each cell, the missing values which have occurred in the pooling process are imputed by their unconditional means.  $\hat{p}_{i}^{(C)}= P\left( T_{i}^{(C)}=1 \vert X_{ij}^{\left( C \right)} \right)=\frac{exp(\beta_{0}^{\left( C \right)}+\sum_{j=1}^{k} \beta_{j}^{\left( C \right)}X_{ij}^{\left( C \right)})}{1+exp(\beta_{0}^{\left( C \right)}+\sum_{j=1}^{k} \beta_{j}^{\left( C \right)}X_{ij}^{\left( C \right)})}$  *Note: Even though the propensity score is estimated in each pooled missingness pattern* ($\hat{p}_{i}^{(C)}$)*, we can use this generalized propensity score* ($\hat{p}_{i}$) *for the analysis.*  **4.** **Estimation of Treatment Effects and its Variance**: Use the propensity score: $\hat{p}_{i}^{(C)}$ (equivalent to $\hat{p}_{i}$) from each pooled cell to compute weighted treatment estimate ($\theta_{MP}$) using inverse probability weighting (IPW) estimator ($\omega_{i}=\frac{1}{\hat{p}_{i}}$ for treated subjects and $\omega_{i}=\frac{1}{1-\hat{p}_{i}}$ for untreated subjects)  $\theta_{MP}=\frac{\sum_{i=1}^{n} \omega_{i}Y_{i}T_{i}}{\sum_{i=1}^{n} \omega_{i}T_{i}}-\frac{\sum_{i=1}^{n} \omega_{i}Y_{i}(1-T_{i})}{\sum_{i=1}^{n} \omega_{i}(1-T_{i})}$  $VAR\left( \theta_{MP} \right)=\frac{1}{n^{2}}\sum_{i=1}^{n} \left( \frac{\omega_{i}(Y_{i}-\hat{\mu}_{T_{i}})}{\sum_{i=1}^{n} \omega_{i}T_{i}} \right)^{2}+\left( \frac{\omega_{i}(Y_{i}-\hat{\mu}_{{(1-T}_{i})})}{\sum_{i=1}^{n} \omega_{i}(1-T_{i})} \right)^{2}$ |

APPENDIX C

Multiple Imputation with Missingness Pattern

| Multiple Imputation with Missingness Pattern (MIMP: Qu & Lipkovich, 2004; Choi et al., 2019; Leite et al., 2021) |
| --- |
| Require: $X_{ij}$ is $n\times p$ (where $i\in n,j\in p$) data matrix containing missing values, $S_{it}$ is the missing pattern indicators (where $i\in n,t\in p$), and $T_{i}$ is the binary treatment indicator and $Y_{i}$ is outcome variable.  **1. Imputation Process**: Generate $M$ complete dataset ($X_{ij}^{(m)}, m=1,2,\ldots, M)$ by imputing missing values in $X_{ij}$ using multiple imputation (e.g., chained equation)   - The imputation process (model) accounts for $T_{i}$, $Y_{i}$, $X_{ij}^{observed}$and $S_{it}$ - Impute missing values $X_{ij}^{missing}\sim P(X_{ij}\vert X_{ij}^{observed},T_{i},S_{it},Y_{i})$   **2. Estimate of propensity score for each imputed dataset**: For each imputed dataset $X_{ij}^{(m)}$, fit a logistic regression model to estimate the propensity score $\hat{p}_{i}^{(m)}$  $\hat{p}_{i}^{(m)}= P\left( T_{i}=1 \vert X_{ij}^{\left( m \right)},S_{ij} \right)=\frac{exp(\beta_{0}^{\left( m \right)}+\sum_{j=1}^{k} \beta_{j}^{\left( m \right)}X_{ij}^{\left( m \right)}+\sum_{t=k+1}^{L} \beta_{t}^{\left( m \right)}S_{it})}{1+exp(\beta_{0}^{\left( m \right)}+\sum_{j=1}^{k} \beta_{j}^{\left( m \right)}X_{ij}^{\left( m \right)}+\sum_{t=k+1}^{L} \beta_{t}^{\left( m \right)}S_{it})}$  Here, $S_{it}$ is included as an additional covariate to adjust for the missingness pattern in estimating propensity score.  **3. Estimate Treatment Effects**: Use the propensity score $\hat{p}_{i}^{(m)}$ from each imputed dataset to compute weighted treatment estimate ($\theta^{(m)}$) using inverse probability weighting (IPW) estimator.  $\theta^{(m)}=\frac{\sum_{i=1}^{n} \omega_{i}^{(m)}Y_{i}T_{i}}{\sum_{i=1}^{n} \omega_{i}^{(m)}T_{i}}-\frac{\sum_{i=1}^{n} \omega_{i}^{\left( m \right)}Y_{i}(1-T_{i})}{\sum_{i=1}^{n} \omega_{i}^{\left( m \right)}(1-T_{i})}$  where $\omega_{i}^{\left( m \right)}=\frac{1}{\hat{p}_{i}^{\left( m \right)}}$ for treated subjects and $\omega_{i}^{\left( m \right)}=\frac{1}{1-\hat{p}_{i}^{\left( m \right)}}$ for untreated subjects.  **4. Combine Treatment Effects and its Variance:** Based on Rubin’s rule, combine the pooled treatment effect estimate ($\hat{\theta}_{MIMP})$ as the mean across the $M$ imputations:  $\hat{\theta}_{MIMP}=\frac{1}{M}\sum_{m=1}^{M} \theta^{(m)}$  And compute the total variance ($T_{var}$) of the pooled estimate by combining within-imputation ($W_{var}$) and between-imputation ($B_{var}$) variances.  $Var_{within}(\hat{\theta}_{MIMP})=\frac{1}{M}\sum_{m=1}^{M} var\left( \theta^{\left( m \right)} \right) , Var_{between}(\hat{\theta}_{MIMP})=\frac{1}{M-1}\sum_{m=1}^{M} \left( \theta^{\left( m \right)}-\bar{\theta} \right)^{2}$  $Var_{total}(\hat{\theta}_{MIMP})=Var_{within}(\hat{\theta}_{MIMP})+\left( 1+\frac{1}{M} \right)Var_{between}(\hat{\theta}_{MIMP})$ |

APPENDIX D

Proximity imputation - Indicator

| Proximity Imputation - Indicator (PI-I; Breiman, 2003) |
| --- |
| Require: $X_{ij}$ is $n\times p$ (where $i\in n,j\in p$) data matrix containing missing values, $S_{it}$ is the missing pattern indicators (where $i\in n,t\in p$), and $T_{i}$ is the binary treatment indicator and $Y_{i}$ is outcome variable.  1. Perform initial imputation by replacing missing values with column medians (Strawman Imputation)  2. Construct a combined data matrix $X^{*}=[X\vert S]$, incorporating both the original data and missing indicators. Then fit the random forest (terminal node: $Y_{i}$) and record how frequently observations share the same terminal nodes in each tree to create a proximity matrix $M$(e.g., If the first and second observation were classified as a same leaf node of one of trees, count one on both (1,2) and (2,1) in the *M* matrix)  3. Convert the frequencies in $M$ into proportions (proximity index):  $M_{ij}^{*}=\frac{M_{ij}}{Total number of trees}$  4. Retain the initial imputed values from Step 1 for subsequent imputation calculations.  5. Calculate the weights based on the proximity index of *M* matrix. If the variable which should be imputed is continuous, it is enough to use proximity index as weights.  $X_{ij}^{missing}=\frac{\sum_{*\in n, *\neq i} X_{*j}M_{i*}^{*}}{\sum_{*\in n, *\neq i} M_{i*}^{*}}$  However, for the categorical variable (e.g., binary), the weights should be calculated by the response categories.  $w_{response = "0"}=\frac{\sum_{*\in n, *\neq i} M_{i*}^{*}I\left( X_{*j}=0 \right))}{\sum_{*\in n, *\neq i} M_{i*}}$  $w_{response = "1"}=\frac{\sum_{*\in n, *\neq i} M_{i*}^{*}I\left( X_{*j}=1 \right))}{\sum_{*\in n, *\neq i} M_{i*}}$  Then, compute the final weight by multiplying these proximity-based weights by the observed category proportions in each row:  $X_{ij}^{missing}=max[\frac{\sum_{j\in p} I\left( X_{*j}=0 \right)}{\#X_{*j}}\times w_{response "0"},\frac{\sum_{j\in p} I\left( X_{*j}=1 \right)}{\#X_{*j}}\times w_{response "1"}]$  The missing value is imputed with the category associated with the larger final  6. Repeat the stage 2 to 5 process until the relative difference of dataset between newly imputed and previous one until reaching small relative difference (1e-5) or the pre-determined reputation times in (5) |

APPENDIX E

Proximity imputation - Unsupervised

| Proximity Imputation - Unsupervised (PI-U; Ishioka, 2012, 2013) |
| --- |
| Require: Require: $X_{ij}$ is $n\times p$ (where $i\in n,j\in p$) data matrix containing missing values, $S_{it}$ is the missing pattern indicators (where $i\in n,t\in p$), and $T_{i}$ is the binary treatment indicator and $Y_{i}$ is outcome variable.  1. Replace missing values in with column medians, creating a "Strawman Imputation" dataset.  2. Construct a combined data matrix $X^{*}=\left[ X \vert S \right]$. Then, an unsupervised random forest algorithm is fitted to $X^{*}$(without explicit outcome $Y_{i}$), generating multiple decision trees. The proximity matrix $M$ is computed by counting how frequently pairs of observations ($i, j$) share the same terminal nodes in each tree.  $M_{ij}=\frac{1}{T}\sum_{t=1}^{T} I(i,j \in leaf_{t})$  3. Change from frequency to percentage within each element (cells) in *M* matrix (Proximity index)  $M_{ij}^{*}=\frac{M_{ij}}{Total number of trees}$  4. Retain the initial imputed values from Step 1 for subsequent imputation calculations.  5. Calculate the weights based on the proximity index of *M* matrix. If the variable which should be imputed is continuous, it is enough to use proximity index as weights.  $X_{ij}^{missing}=\frac{\sum_{*\in n, *\neq i} X_{*j}M_{i*}^{*}}{\sum_{*\in n, *\neq i} M_{i*}^{*}}$  However, for the categorical variable (e.g., binary), the weights should be calculated by the response categories.  $w_{response = "0"}=\frac{\sum_{*\in n, *\neq i} M_{i*}^{*}I\left( X_{*j}=0 \right))}{\sum_{*\in n, *\neq i} M_{i*}}$  $w_{response = "1"}=\frac{\sum_{*\in n, *\neq i} M_{i*}^{*}I\left( X_{*j}=1 \right))}{\sum_{*\in n, *\neq i} M_{i*}}$  Then, compute the final weight by multiplying these proximity-based weights by the observed category proportions in each row:  $X_{ij}^{missing}=max[\frac{\sum_{j\in p} I\left( X_{*j}=0 \right)}{\#X_{*j}}\times w_{response "0"},\frac{\sum_{j\in p} I\left( X_{*j}=1 \right)}{\#X_{*j}}\times w_{response "1"}]$  The missing value is imputed with the category associated with the larger final  6. Repeat the stage 2 to 5 process until the relative difference of dataset between newly imputed and previous one until reaching small relative difference (1e-5) or the pre-determined reputation times in (5) |

APPENDIX F. The number of pre-pooling distinct pattern per manipulated condition


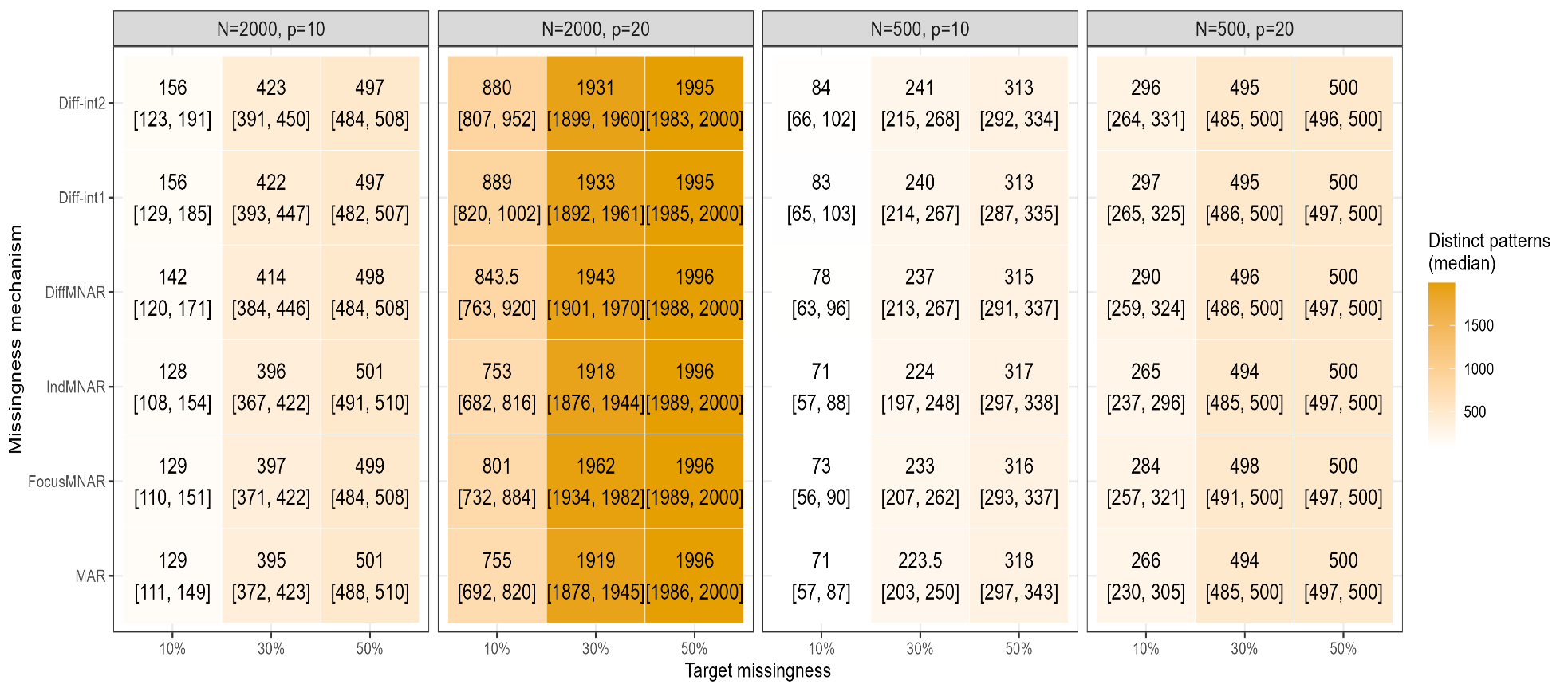


*APPENDIX F presents that the number of distinct missingness patterns (pre-pooling) is strongly driven by the percentage of missingness (N) and the number of covariates (p). When the missingness is low and the number of covariates is small, the number of missingness patterns is modest, whereas with higher percentage of missingness (over 30%) and larger number of covariates, the number of patterns drastically increases and can reach to the sample size, indicating many sparse pattern strata.*

APPENDIX G. Post-pooling diagnostics: minimum pooled-group size and proportion of groups below 100


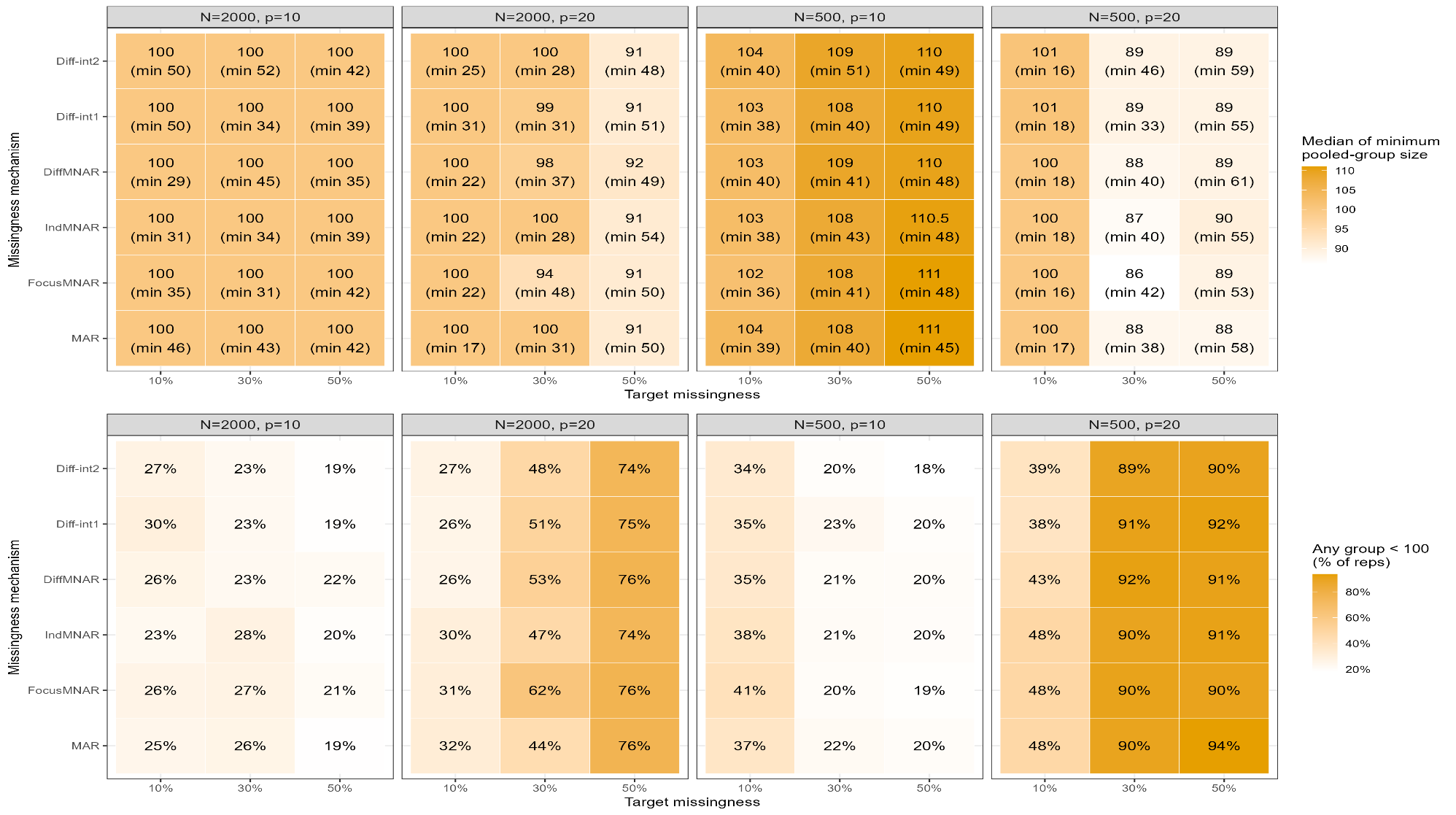


*APPENDIX G confirms that the “100” observations functions as a minimum target cell-size threshold used in the pooling step rather than an exact pattern size: The median of minimum pooled group size is near 100 in many conditions, but the minimum across replications can be smaller in some settings, and the proportion of replications with any pooled group in which at least one pooled group remained below 100 can be non-negligible. Together, these figures document the data-driven nature of missingness patterns and the extent to which pooling is required to stabilize pattern-based estimation.*
